# Protective Effects of Prenatal *Bacopa monnieri* Extract Against Valproic Acid-Induced Autism-like Behavioral and Neurohistological Alterations in Mice

**DOI:** 10.3390/molecules31162783

**Published:** 2026-08-10

**Authors:** Zainab M. Almalki, Ashwaq H. Batawi, Asma Almuhammadi, Safaa A. Alowaidi, Suad H. Almasoudi, Jehan Alamri, Mona A. AL-Thepyani

**Affiliations:** 1 Department of Biological Science, Faculty of Sciences, King Abdulaziz University, P.O. Box 80200, Jeddah 21589, Saudi Arabia; zalmalki0032@stu.kau.edu.sa (Z.M.A.); amalmuhammadi@kau.edu.sa (A.A.); saalowadhi@kau.edu.sa (S.A.A.); jsalamri@kau.edu.sa (J.A.); 2 Department of Biology, College of Sciences, Umm Al-Qura University, Makkah 21955, Saudi Arabia; shmasuadi@uqu.edu.sa; 3 Department of Chemistry, College of Sciences & Arts, King Abdulaziz University, Rabigh 21911, Saudi Arabia; mahalthepyani@kau.edu.sa; 4 Neuroscience and Geroscience Research Unit, King Fahd Medical Research Center, King Abdulaziz University, Jeddah 21589, Saudi Arabia

**Keywords:** autism spectrum disorder, *Bacopa monnieri*, valproic acid, oxidative stress, hippocampus, cerebellum

## Abstract

Autism spectrum disorder (ASD) is a neurodevelopmental condition characterized by impaired social communication and repetitive behaviors. Oxidative stress is increasingly recognized as a key contributor to the neuro-degeneration and behavioral abnormalities associated with ASD. *Bacopa monnieri* (BM), a medicinal herb with potent antioxidant and neuro-protective properties, has shown promise in mitigating oxidative damage. This study evaluated the preventive effects of BM extract on behavioral and neuro-histological alterations in a valproic acid (VPA)-induced mouse model of ASD. Pregnant mice received BM extract (400 mg/kg, orally) throughout gestation, while VPA (600 mg/kg) was administered intraperitoneally on embryonic day 12 (E12). Behavioral assessments included the open field test, righting reflex, three-chamber social interaction, marble burying, and hot plate tests. Oxidative stress markers, malondialdehyde (MDA) and glutathione (GSH), were quantified in hippocampal and cerebellar tissues. BM-treated offspring showed significant behavioral improvements, including reduced hyperactivity in the open field test (*p* < 0.0001), along with restored brain tissue architecture. Moreover, BM extract decreased MDA levels and elevated GSH concentrations, indicating attenuation of oxidative stress. In conclusion, *Bacopa monnieri* extract exerts protective effects against autism-like symptoms by enhancing antioxidant defenses and preserving neural integrity, suggesting its potential as a natural preventive strategy for ASD.

## 1. Introduction

Autism Spectrum Disorder (ASD) is a complex neurodevelopmental condition characterized by persistent deficits in social communication and interaction, accompanied by restricted, repetitive patterns of behavior, interests, or activities [1]. Over recent decades, the global prevalence of ASD has risen markedly, with current estimates indicating that approximately one in every 100 children is diagnosed with the disorder [2]. Although the precise etiology of ASD remains unclear, converging evidence suggests that genetic, environmental, and epigenetic factors collectively contribute to its pathogenesis [3,4].

Emerging studies have underscored the pivotal role of neuroinflammation and oxidative stress in ASD, both of which contribute to synaptic dysfunction and neurotransmitter imbalance [5,6]. Notably, disturbances in glutamatergic and GABAergic neurotransmission have been reported in individuals with ASD, reflecting an imbalance between excitatory and inhibitory signaling in critical brain regions such as the hippocampus and cerebellum [7]. These neurochemical disruptions are believed to underlie many of the sensory processing abnormalities and cognitive impairments characteristic of ASD [8]. Furthermore, elevated levels of reactive oxygen species (ROS) and diminished antioxidant defenses—evidenced by decreased glutathione (GSH) and increased malondialdehyde (MDA) concentrations—have been documented in both plasma and brain tissues of ASD patients [9,10]. These findings indicate a strong association between redox imbalance, mitochondrial dysfunction, and neuronal vulnerability in ASD.

Several medications administered during pregnancy have been associated with an increased risk of ASD in offspring, among which valproic acid (VPA) is one of the most extensively studied [11]. VPA has been shown to alter gene expression, inhibit histone deacetylase (HDAC) activity, and disrupt neurogenesis when administered during critical periods of neural tube development [3]. These molecular and developmental alterations, which have been consistently replicated in animal models, lead to behavioral abnormalities in offspring—particularly repetitive behaviors and social interaction deficits—that closely mimic core features of ASD [12].

Currently available pharmacological treatments for ASD primarily aim to manage associated symptoms such as irritability, hyperactivity, and repetitive behaviors rather than addressing the core deficits in social communication or cognition. The most commonly prescribed medications, including atypical antipsychotics such as risperidone and aripiprazole, have shown partial efficacy but are often accompanied by significant adverse effects, including weight gain, metabolic disturbances, sedation, and extrapyramidal symptoms [13,14]. Stimulant medications and selective serotonin reuptake inhibitors (SSRIs), used to treat hyperactivity and anxiety in ASD, also exhibit variable efficacy and may lead to increased irritability, sleep disturbances, and gastrointestinal issues [15,16]. Furthermore, the long-term safety and tolerability of these pharmacotherapies in children remain uncertain [17]. Given these limitations and side effects, there is a growing interest in exploring alternative and complementary therapeutic strategies, including the use of natural compounds with neuroprotective and antioxidant potential.

In recent years, increasing attention has been directed toward the use of natural products as potential therapeutic agents for neurodevelopmental disorders, including ASD. Conventional pharmacological treatments for ASD are limited in efficacy and often accompanied by undesirable side effects, highlighting the need for safer and more effective alternatives [18]. Natural compounds derived from medicinal plants contain diverse bioactive constituents with antioxidant, anti-inflammatory, and neuroprotective activities that can modulate multiple pathological mechanisms involved in ASD [5,19]. By restoring redox homeostasis, attenuating neuroinflammation, and regulating neurotransmitter systems, these phytochemicals offer a promising multi-target approach to ameliorate ASD-related behavioral and neurochemical disturbances [20]. Consequently, the exploration of natural products as potential therapeutic or preventive interventions represents an emerging and valuable direction in ASD research [21].

Among the natural herbal candidates explored for their neuroprotective potential, *Bacopa monnieri* (L.) Pennell, commonly known as Brahmi in Ayurvedic medicine, has attracted considerable scientific attention. This medicinal herb is rich in bioactive constituents, particularly bacosides A and B, which exhibit potent antioxidant, anti-inflammatory, and neuromodulatory properties [22]. Both clinical and preclinical investigations have demonstrated that *B. monnieri* enhances memory, attention, and cognitive performance, while also alleviating anxiety, depression, and sleep disturbances [23]. Owing to its ability to modulate neurotransmitter systems, reduce oxidative stress, and support mitochondrial integrity, *B. monnieri* has emerged as a promising candidate for the management of neurodevelopmental disorders, including ASD [24].

To date, no study has examined the preventive efficacy of *B. monnieri* when administered throughout gestation in a VPA-induced model of ASD. Although its neuroprotective effects have been well-documented in various experimental paradigms, its potential as a maternal intervention during pregnancy to prevent ASD-like phenotypes in offspring remains unexplored.

In this study, we hypothesized that prenatal administration of *B. monnieri* extract (BME) throughout pregnancy could confer neuroprotective effects against VPA-induced ASD-like behaviors in offspring. These effects were expected to manifest as improved behavioral outcomes, preserved brain histoarchitecture, and reduced oxidative stress. To our knowledge, this is the first study to evaluate the prophylactic potential of continuous maternal administration of *B. monnieri* during the entire gestational period in a VPA-induced ASD mouse model. This innovative approach may offer valuable insights into the potential of early maternal nutritional or phytotherapeutic interventions to mitigate ASD risk in offspring.

## 2. Results

### 2.1. Major Characterized Constituents of Bacopa monnieri Extract

**Figure 1:**
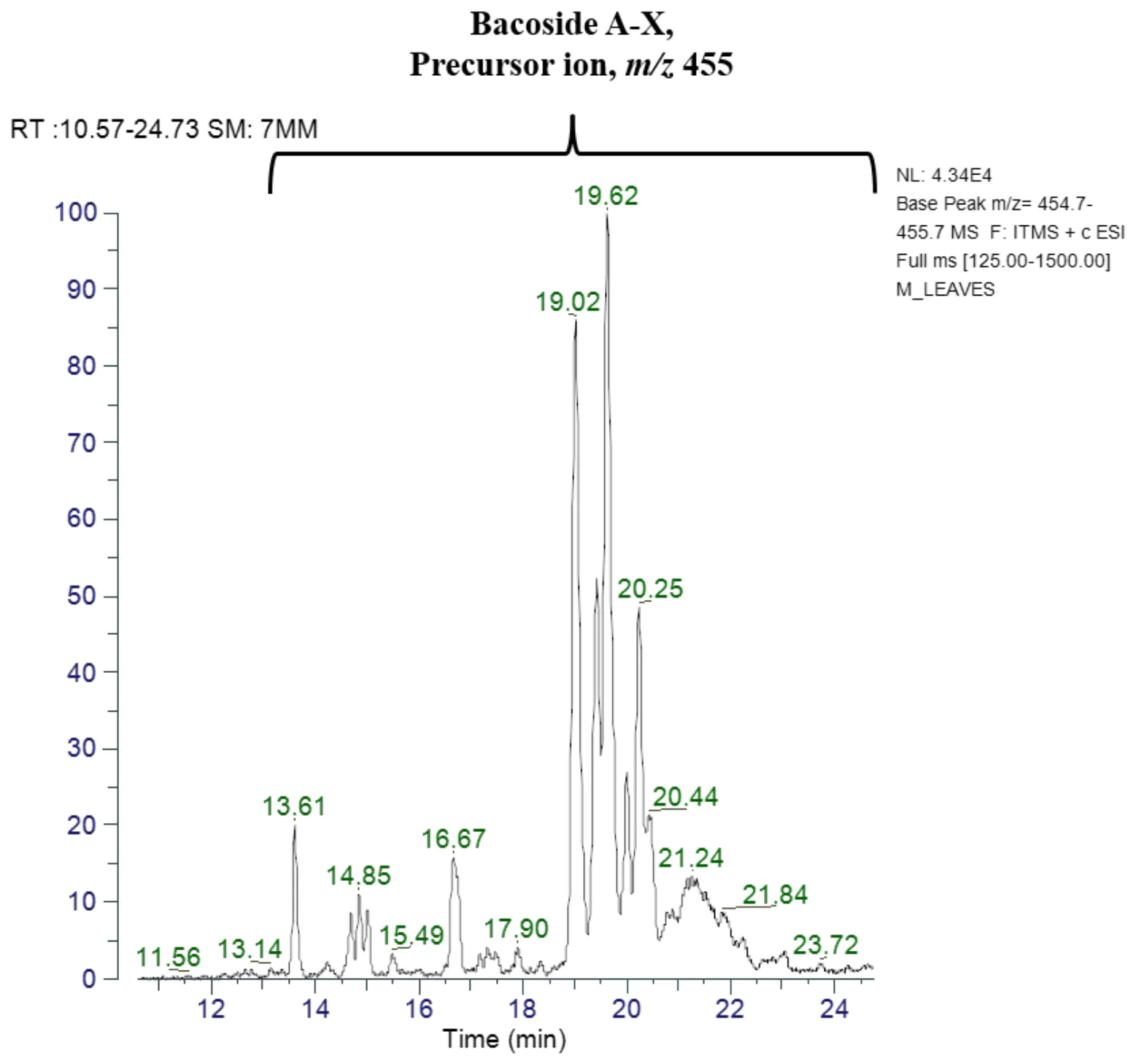
Extracted Ion Chromatogram (EIC) of *m*/*z* 455, the characteristic precursor positive ESI ion of Bacoside A to X compounds in *Bacopa monnieri* extract.

**Figure 2:**
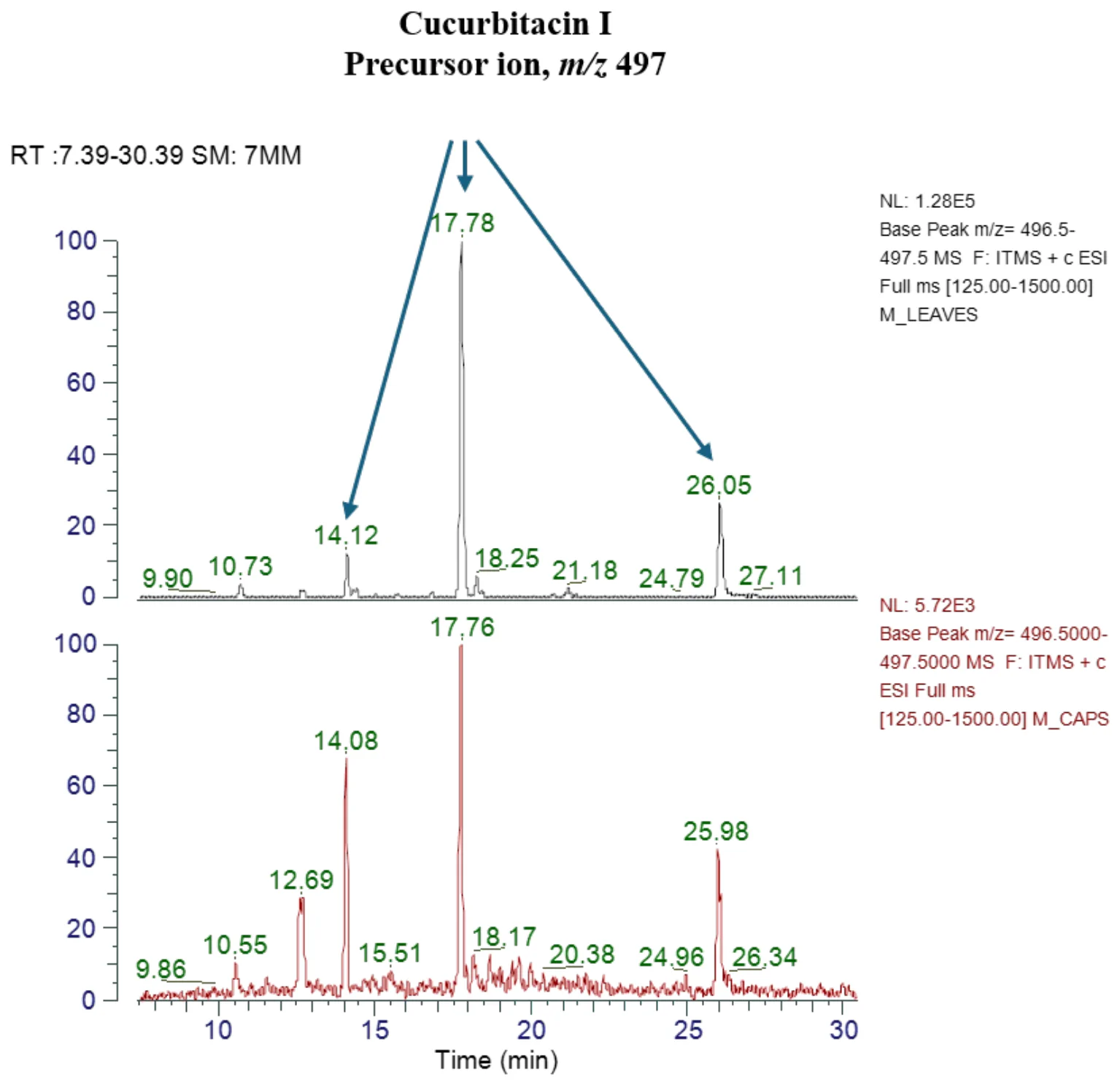
Extracted Ion Chromatogram (EIC) of *m*/*z* 497 the characteristic precursor positive ESI ion of Cucurbitacin compounds in *Bacopa monnieri* extract.

LC-ESI-Ion Trap MS analysis revealed that the *Bacopa monnieri* extract contained a diverse range of phytochemicals, including triterpenoid saponins, triterpenes, flavonoids, phenolic glycosides, and fatty acid derivatives (Table 1). The predominant constituents were bacosides (Figure 1), detected over a retention time range of 13.6–21.2 min, including the characteristic compound bacoside A (Rt = 26.8 min), which is considered the principal bioactive constituent responsible for many of the neuroprotective, cognitive-enhancing, and antioxidant properties of *B. monnieri*. Other major triterpenoid constituents included cucurbitacin I (Figure 2), detected at multiple retention times (14.12, 17.78, and 26.06 min), senegenin, and esculentic acid. The extract also contained several flavonoids and their glycosides, including luteolin, luteolin 7-glucuronide, and genistein-4′-glucuronide, which are known for their antioxidant and anti-inflammatory activities. In addition, phenolic glycosides such as echinacoside and 6′-(p-hydroxybenzoyl)mussaenosidic acid, together with the oxylipin (9Z,12E)-15,16-dihydroxyoctadeca-9,12-dienoic acid, were identified. The presence of these bioactive compounds suggests that the pharmacological effects of the *B. monnieri* extract are likely attributable to the synergistic actions of multiple phytochemical constituents rather than a single active compound.

#### 2.1.1. Effect of BME Against VPA-Induced Developmental Abnormalities

**Figure 3:**
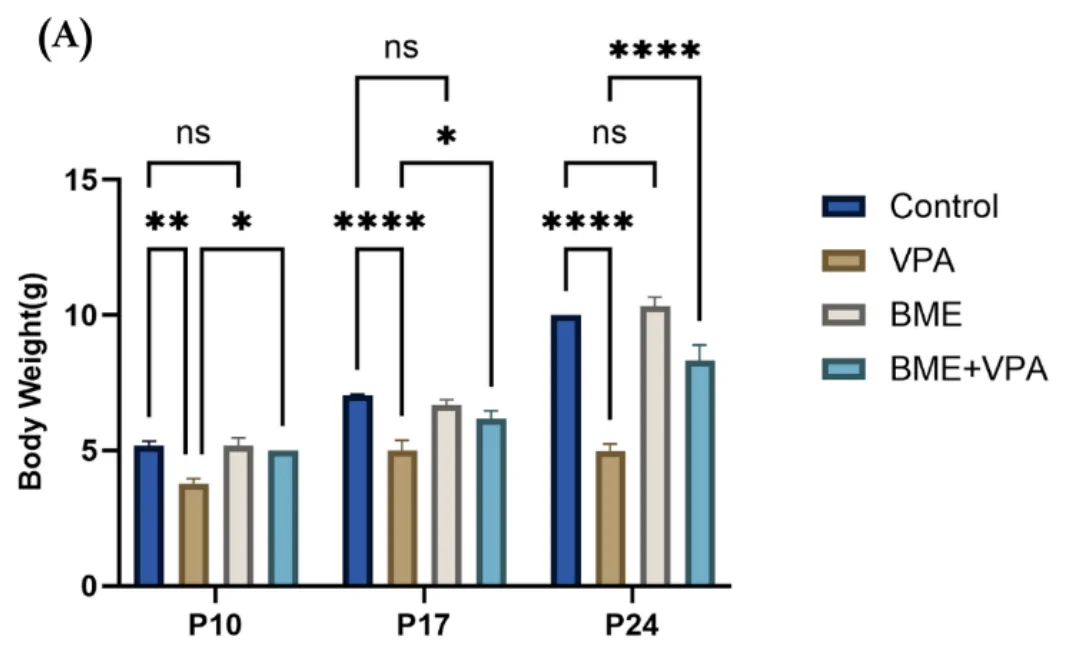

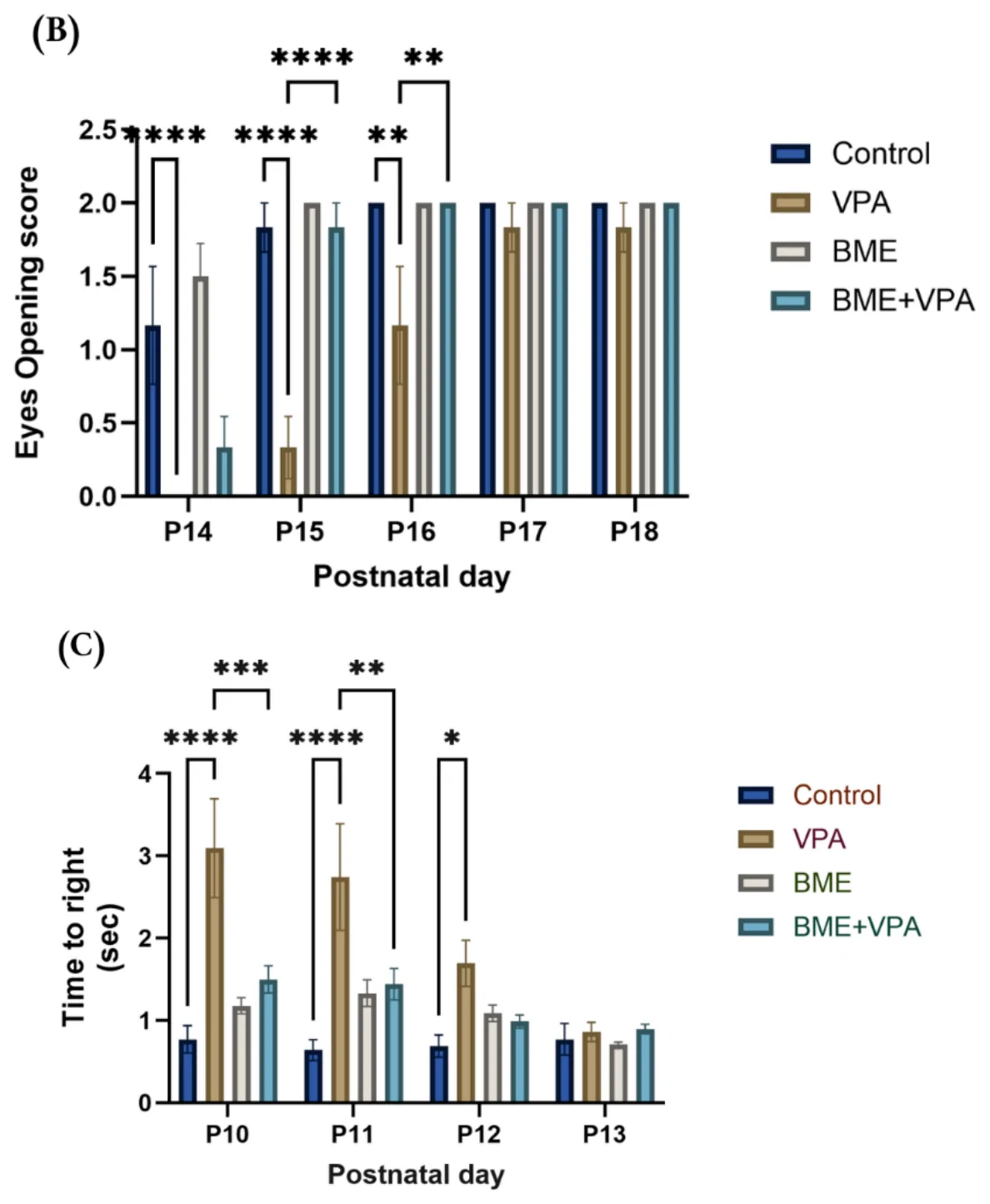
Developmental delay in mice prenatally exposed to VPA. (**A**) Body weight at P10, P17, and P24. (**B**) Eye-opening scores (P14–P18). (**C**) Righting reflex latency (P10–P13). Data are presented as mean ± SEM (n = 6 per group). Asterisk indicates statistical significance: * *p* < 0.05, ** *p* < 0.01, *** *p* < 0.001, **** *p* < 0.0001. Data were analyzed using one-way or two-way ANOVA followed by Tukey’s post hoc test, ns: non-significant.

Prenatal VPA exposure produced clear developmental abnormalities, with pups showing reduced body size compared to controls. However, co-treatment with BME (400 mg/kg) partially improved growth outcomes. Body weight analysis proved that VPA-exposed pups showed significantly lower body weight than controls at postnatal days (P10, P17, and P24), whereas the BME + VPA group exhibited significantly higher weights than the VPA group at the same time points. No differences were detected between control and BME-only groups (Figure 3A). Litter size appeared reduced in the VPA group, but the difference was not statistically significant compared with control group (*p* = 0.6945).

The eye-opening test indicated that VPA-exposed pups showed significantly reduced scores at P14–P16 compared with controls. BME + VPA pups demonstrated significant improvement at P15–P16, while no changes were observed at other time points (Figure 3B).

Righting reflex performance revealed longer righting latency in VPA pups at P10–P12, whereas BME + VPA pups showed significantly shorter latency at P10–P11 compared to the VPA group. Control and BME-only groups did not differ significantly (Figure 3C).

#### 2.1.2. Role of BME Against Motor Deficits Induced by VPA

**Figure 4:**
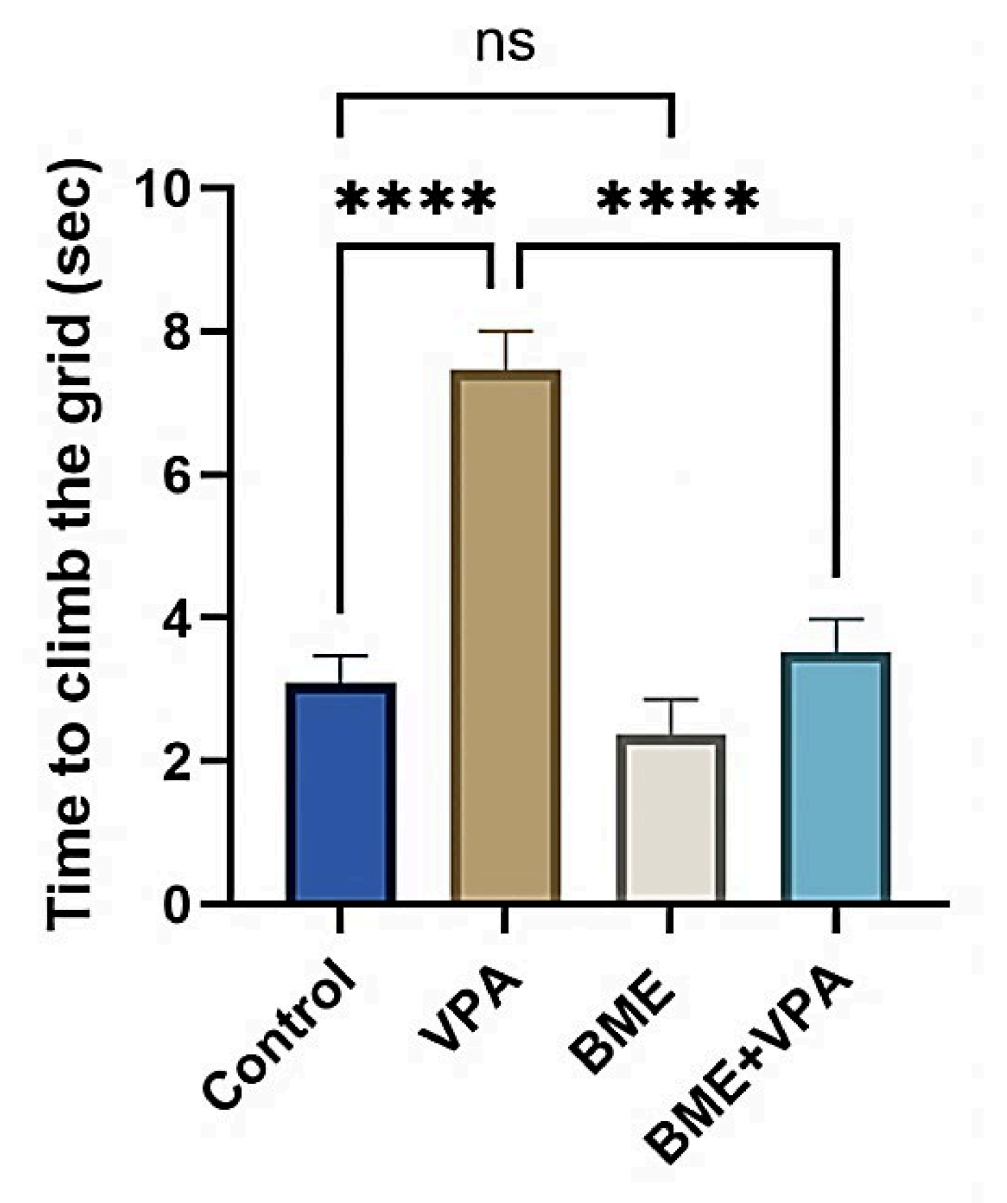
Effect of BME on motor deficits in mice prenatally exposed to VPA. VPA-exposed pups exhibited delayed motor coordination in the negative geotaxis test, whereas BME + VPA treatment improved performance. Data are presented as mean ± SEM (n = 6 per group). Statistical significance is indicated as **** *p* < 0.0001. Analyses were performed using one-way ANOVA followed by Tukey’s post hoc test, ns: non-significant.

Motor function was evaluated using the negative geotaxis test (Figure 4). VPA-exposed pups required significantly more time to reorient and climb upward compared to controls (*p* < 0.0001), indicating impaired motor coordination. No significant differences were observed between the control and BME-only groups. Importantly, BME + VPA treatment significantly reduced climbing time relative to the VPA group (*p* < 0.0001), demonstrating improved motor performance and partial neuroprotection.

#### 2.1.3. BME Impact Against VPA-Induced Social Cognitive Dysfunction

**Figure 5:**
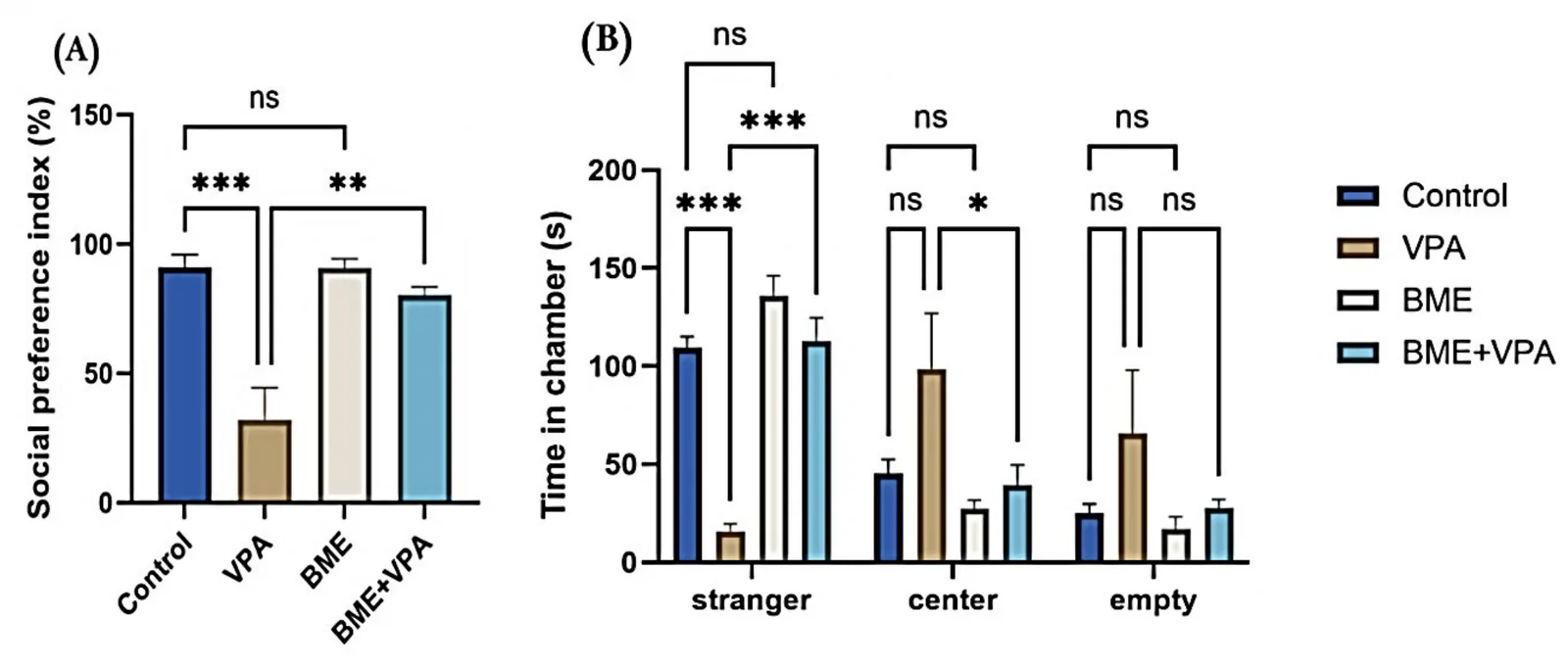
Effect of BME on social interactions in mice prenatally exposed to VPA. (**A**) Social preference index. (**B**) Time spent in each chamber during the three-chamber test. Data are presented as mean ± SEM (n = 6 per group). Statistical significance is indicated as * *p* < 0.05, ** *p* < 0.01, *** *p* < 0.001. One-way ANOVA (social preference index) and two-way repeated-measures ANOVA (time in chambers) were used, followed by Tukey’s multiple comparisons test, ns: non-significant.

Social cognition was assessed using the three-chamber test. As shown in Figure 5A, VPA-exposed mice exhibited a significantly reduced social preference index compared to controls (*p* = 0.0004), indicating marked sociability deficits. No difference was observed between the control and BME groups (*p* > 0.9999). The BME + VPA group showed a significant improvement compared with the VPA group (*p* = 0.0021); however, the social preference index did not fully return to control levels, indicating a partial restoration of social behavior.

Time spent in each chamber (Figure 5B) supported these findings. Control mice spent significantly more time in the stranger chamber than in the empty chamber (*p* = 0.0004), reflecting normal social preference. VPA-exposed mice lacked this preference, spending less time in the stranger chamber and more time in the center and empty chambers. BME alone produced no changes relative to controls. Importantly, BME + VPA treatment significantly increased time spent in the stranger chamber (*p* = 0.0003) and center chamber (*p* = 0.0333) compared with VPA, with no significant change in the empty chamber (*p* = 0.2594). However, the time spent in the stranger chamber did not fully return to the level observed in the control group, indicating a partial recovery of social interaction.

#### 2.1.4. Neuroprotective Effect of BME Against VPA-Induced Repetitive and Stereotypic Behaviors

**Figure 6:**
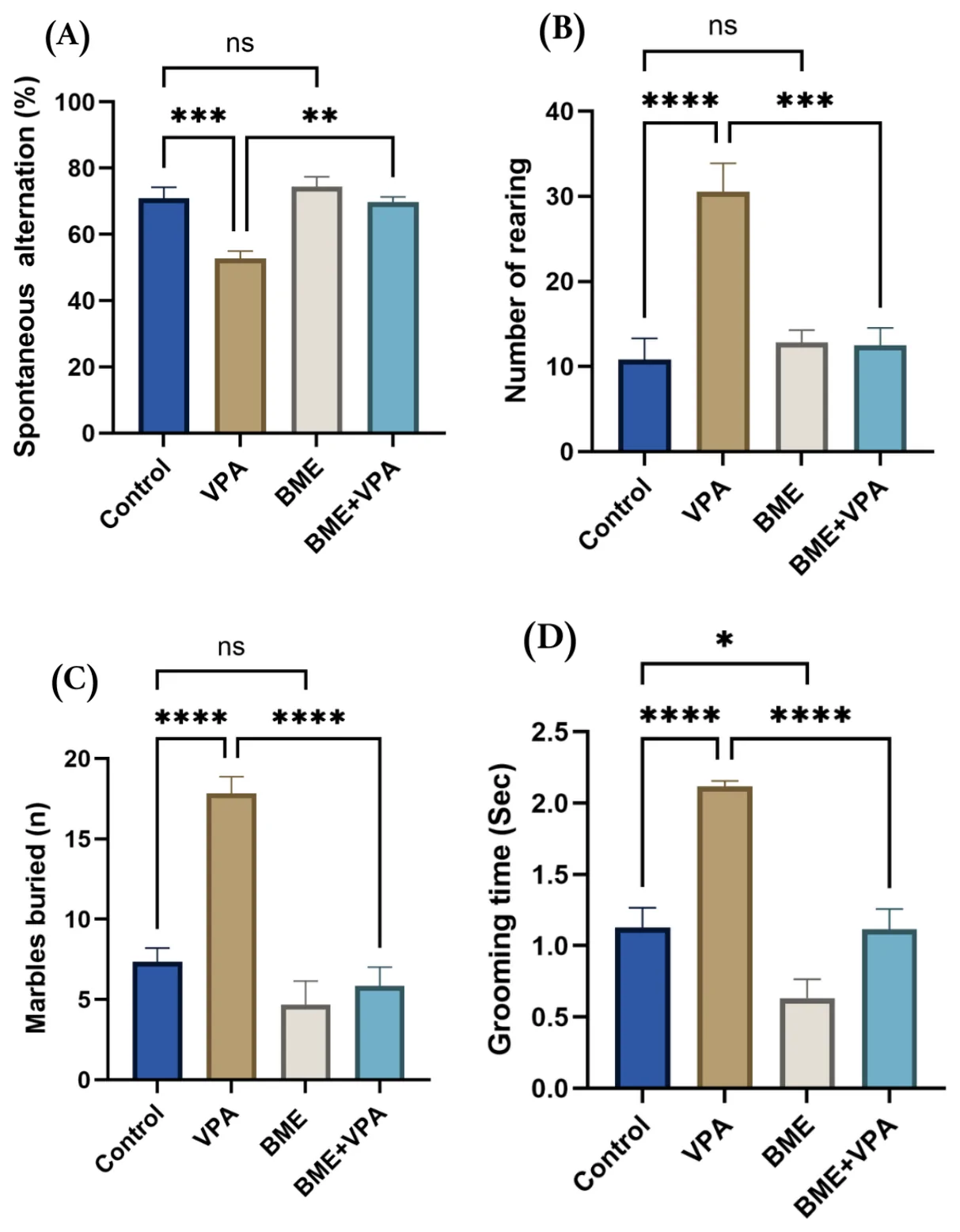
Effect of BME on repetitive behaviors in mice prenatally exposed to VPA. Repetitive and stereotypic behaviors were assessed using (**A**) Y-maze test, (**B**) cylinder test, (**C**) marble burying test, and (**D**) splash test. Data are presented as mean ± SEM (n = 6 per group). Statistical significance is indicated as * *p* < 0.05, ** *p* < 0.01, *** *p* < 0.001, **** *p* < 0.0001. Analyses were performed using one-way ANOVA followed by Tukey’s post hoc test, ns: non-significant.

Repetitive and compulsive-like behaviors were assessed using multiple behavioral tests. In the Y-maze test (Figure 6A), VPA-exposed mice showed a significantly reduced spontaneous alternation percentage compared to controls (*p* = 0.0006), indicating impaired cognitive flexibility. The BME + VPA group displayed significant improvement (*p* = 0.0012), while BME alone produced no changes.

In the cylinder test (Figure 6B), VPA-exposed mice exhibited a marked increase in rearing behavior (*p* < 0.0001). No differences were found between control and BME groups, whereas BME + VPA treatment significantly reduced rearing compared to VPA (*p* = 0.0002).

In the marble burying test (Figure 6C), VPA-exposed mice buried significantly more marbles than controls (*p* < 0.0001), reflecting heightened repetitive behavior. BME alone showed no effect, while BME + VPA significantly decreased marble burying compared to VPA (*p* < 0.0001).

In the splash test (Figure 6D), VPA-exposed mice spent significantly more time grooming than controls (*p* < 0.0001). The BME group showed a slight increase compared to controls (*p* = 0.0395). Notably, BME + VPA treatment significantly reduced grooming duration relative to VPA (*p* < 0.0001), indicating partial recovery.

#### 2.1.5. Effect of BME Against VPA-Induced Hyperactivity

**Figure 7:**
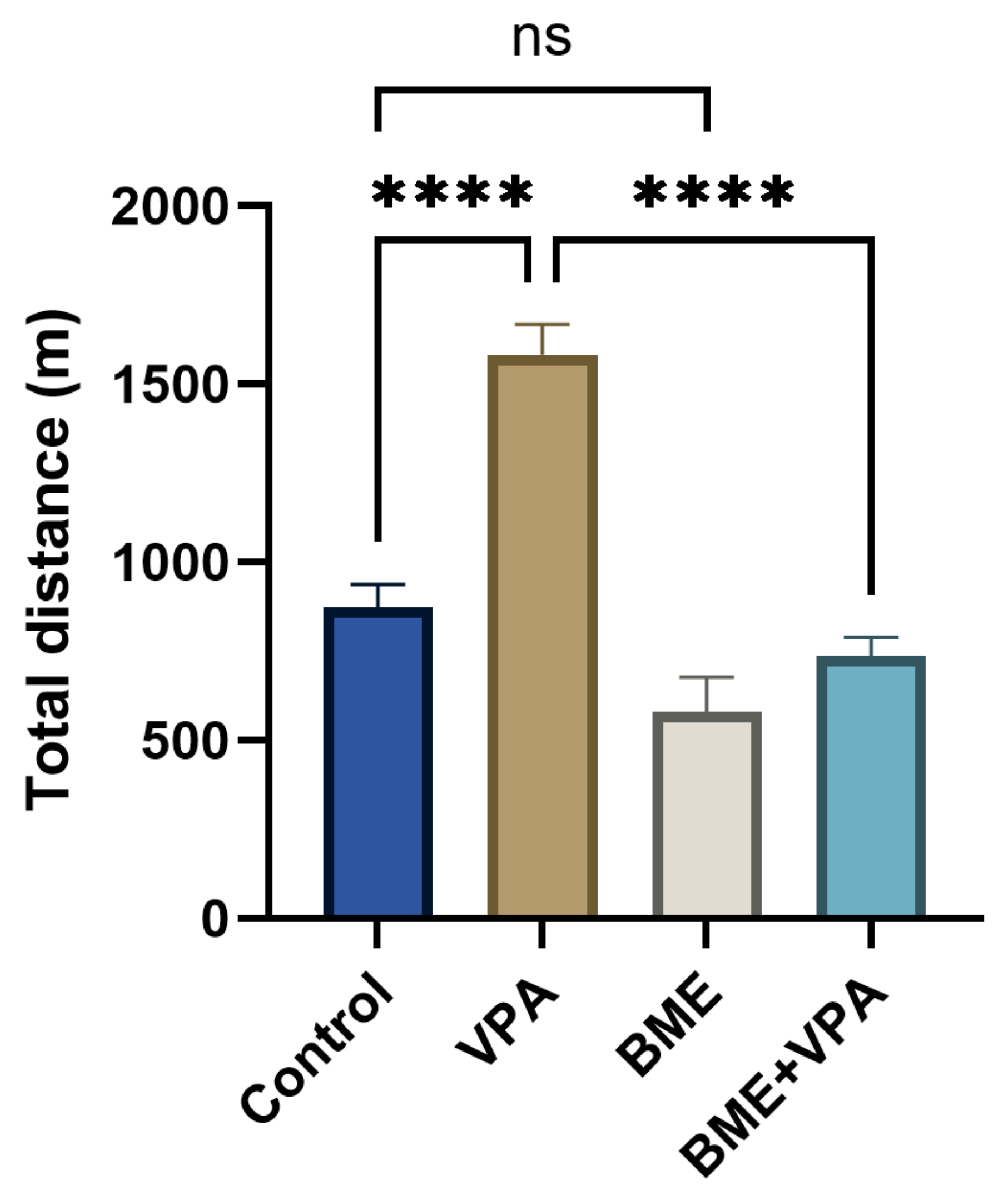
Effect of BME on hyperactivity in mice prenatally exposed to VPA. Locomotor activity was assessed using the open field test. Data are presented as mean ± SEM (n = 6 per group). Statistical significance is indicated as **** *p* < 0.0001. Analysis was performed using one-way ANOVA followed by Tukey’s multiple comparisons test, ns: non-significant.

Locomotor activity was evaluated using the open field test by measuring total distance traveled (Figure 7). VPA-exposed mice showed significantly increased locomotion compared to controls (*p* < 0.0001), indicating prenatal VPA-induced hyperactivity. No significant differences were observed between control and BME-only groups. Remarkably, BME + VPA treatment significantly reduced total distance traveled relative to the VPA group (*p* < 0.0001), demonstrating a protective effect of BME against hyperactive behavior.

#### 2.1.6. Neuroprotective Potential of BME Against VPA-Induced Sensory Abnormalities

**Figure 8:**
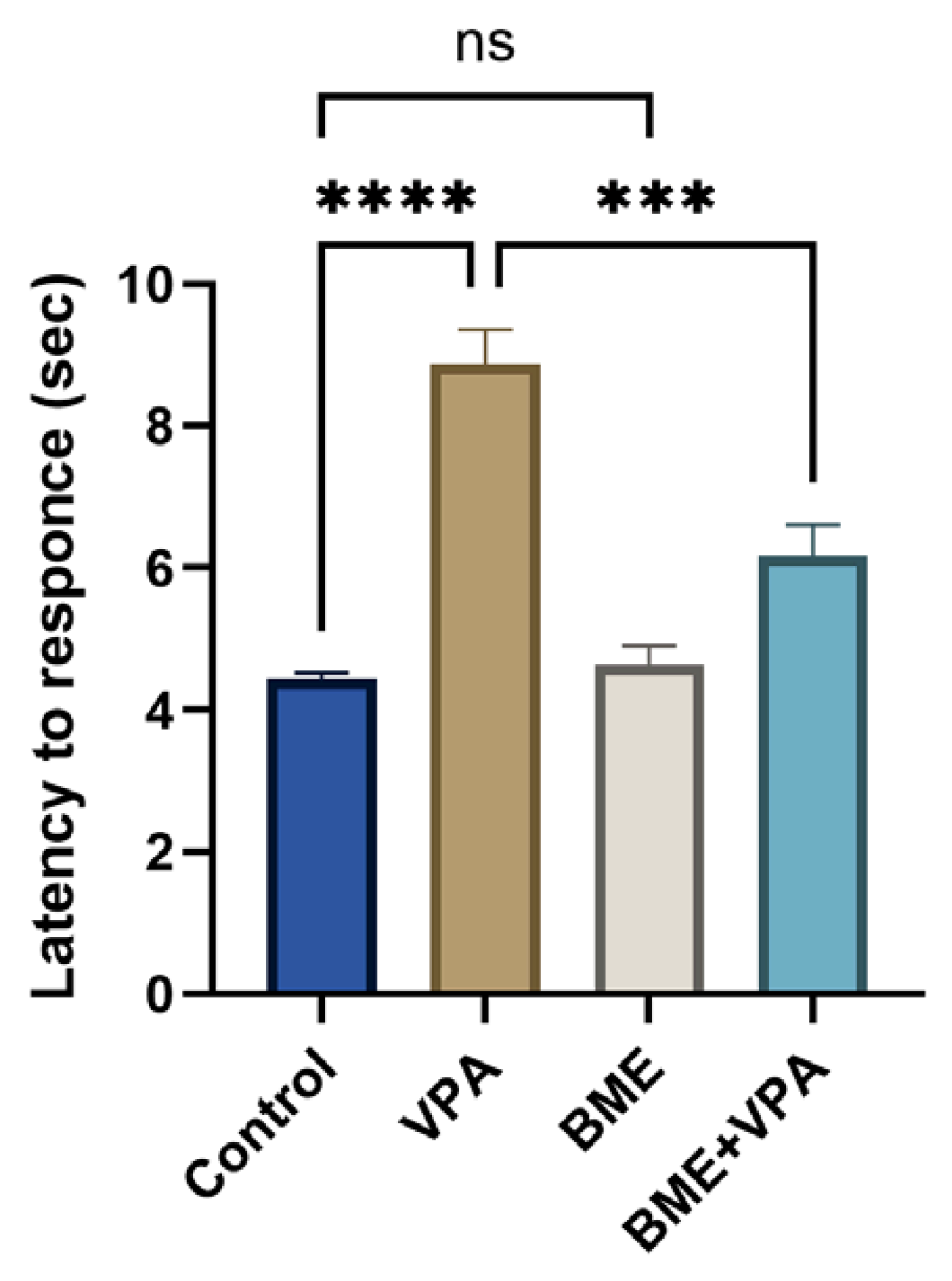
Effect of BME on sensory responses in mice prenatally exposed to VPA, assessed by the hot plate test. Data are presented as mean ± SEM (n = 4 per group). Statistical significance is indicated as *** *p* < 0.001, **** *p* < 0.0001. Analyses were performed using one-way ANOVA followed by Tukey’s post hoc test, ns: non-significant.

Nociceptive responses were assessed using the hot plate test (Figure 8). VPA-exposed mice exhibited significantly increased response latency compared to controls (*p* < 0.0001), indicating impaired sensory perception. No differences were observed between control and BME-only groups. Importantly, BME + VPA treatment significantly reduced response latency relative to the VPA group (*p* = 0.0008), suggesting a partial protective effect on sensory function.

### 2.2. Biochemical Results

#### Alterations in Brain MDA and GSH Levels

MDA and GSH were measured as markers of oxidative stress. VPA exposure significantly elevated MDA levels compared to the control group (*p* = 0.0014), while BME + VPA treatment markedly reduced these levels (*p* = 0.0247) compared to VPA group. However, no significant difference was observed between the control and BME groups (Table 2).

Conversely, GSH levels were significantly decreased in the VPA group (*p* = 0.0032) compared to control group. BME + VPA treatment improved GSH levels compared to VPA alone (*p* = 0.0342). However, no significant differences were observed between the control and BME groups (Table 3).

### 2.3. Histological Examination

Histological analysis focused on the hippocampus (particularly the dentate gyrus) and the cerebellum, including the molecular, Purkinje, and granular layers, to provide a comprehensive assessment of cortical organization.

#### 2.3.1. Histological Changes in the Hippocampus of the Dentate Gyrus

**Figure 9:**
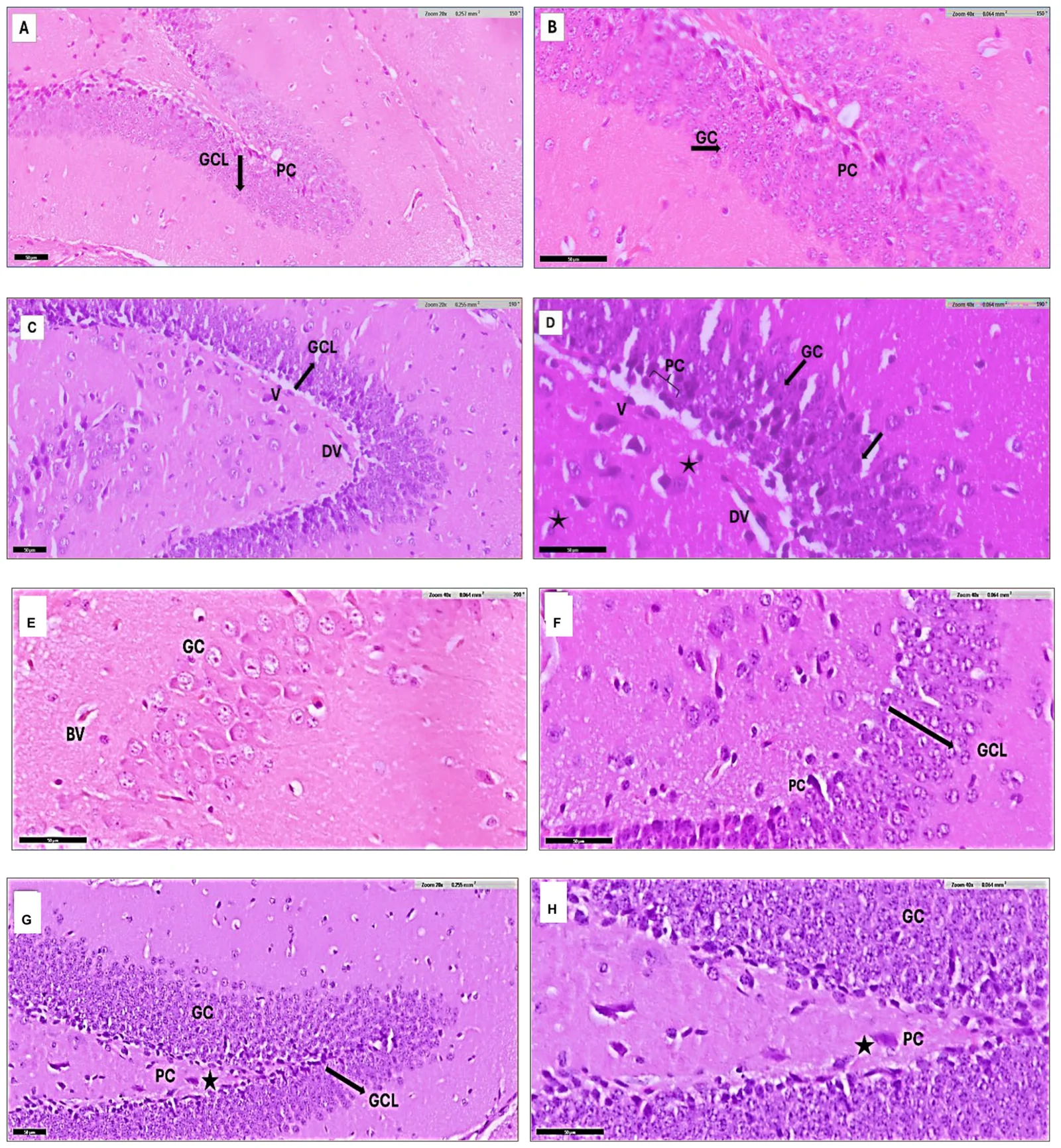
Photomicrographs of H&E-stained hippocampal sections showing the dentate gyrus (DG) region in the control group (**A**,**B**), VPA group (**C**,**D**), BME group (**E**,**F**) and BME + VPA group (**G**,**H**). Granular cell layer: black arrow GCL, Pyramidal cells: PC, Vacuoles: V, Dilated blood vessels: DV.

The dentate gyrus of the control group (Figure 9A) exhibited normal histological architecture, with a densely packed granular cell layer composed of rounded granule cells and intact pyramidal neurons. Higher magnification (Figure 9B) confirmed the compact arrangement of granular cells with large, deeply stained nuclei, along with normal-appearing pyramidal cells. In the VPA group, the dentate gyrus exhibited marked structural disruption, with a disorganized and less dense granular cell layer, numerous vacuoles, and noticeably dilated blood vessels (Figure 9C). Higher magnification revealed irregular granular cells, abnormal pyramidal cell morphology, and shrunken cells with pyknotic nuclei (Figure 9D). In the BME-treated group, the dentate gyrus displayed preserved histological architecture, with compact, rounded granular cells and intact blood vessels (Figure 9E). The granular layer remained well organized, and both granular and pyramidal cells maintained normal morphology (Figure 9F). In the BME + VPA group, the dentate gyrus retained a largely preserved architecture, with a densely packed and well-organized granular cell layer and intact blood vessels (Figure 9G). Pyramidal cells showed early signs of morphological recovery, while granular cells maintained their normal arrangement (Figure 9H).

#### 2.3.2. Histological Changes in the Cerebellum

**Figure 10:**
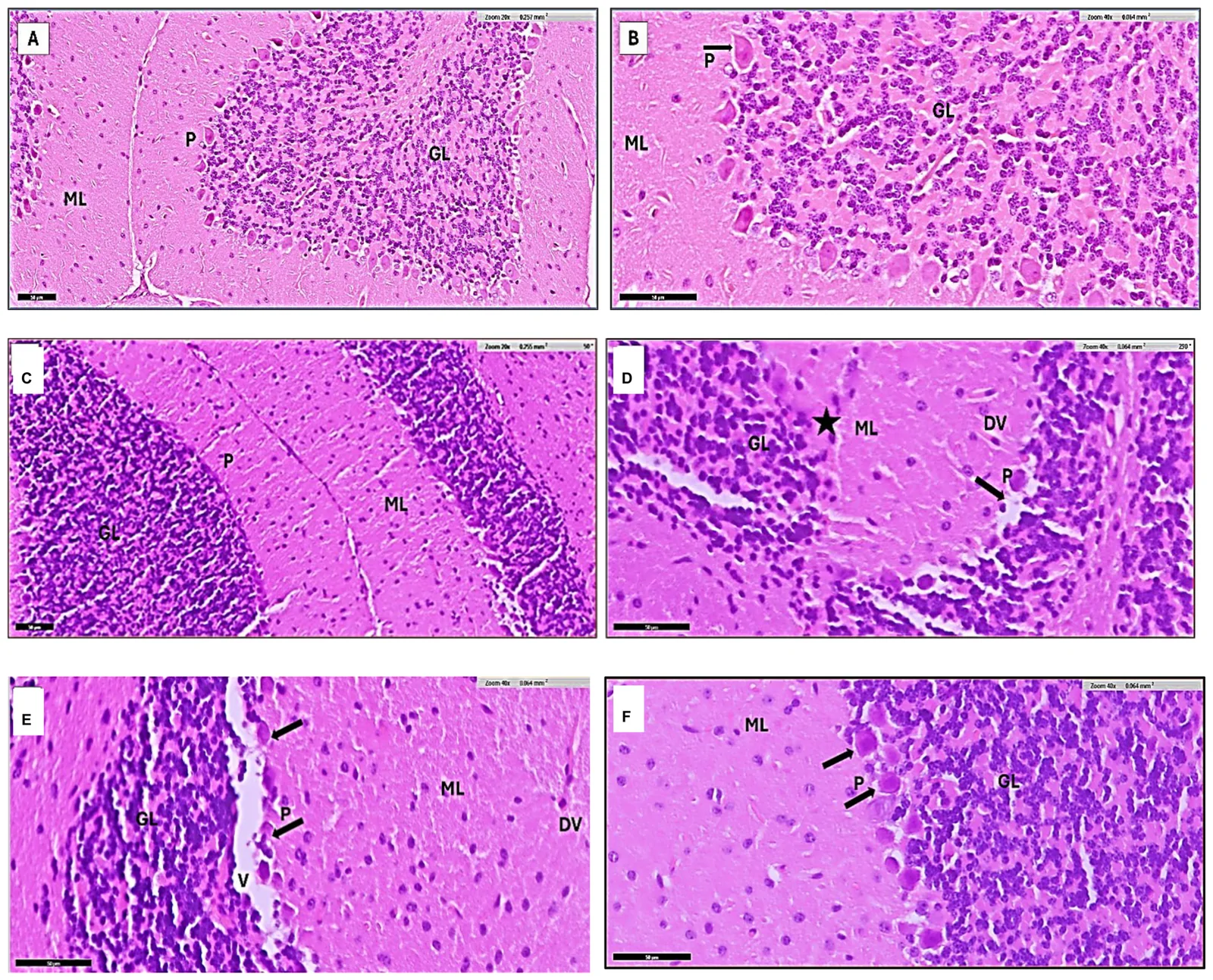

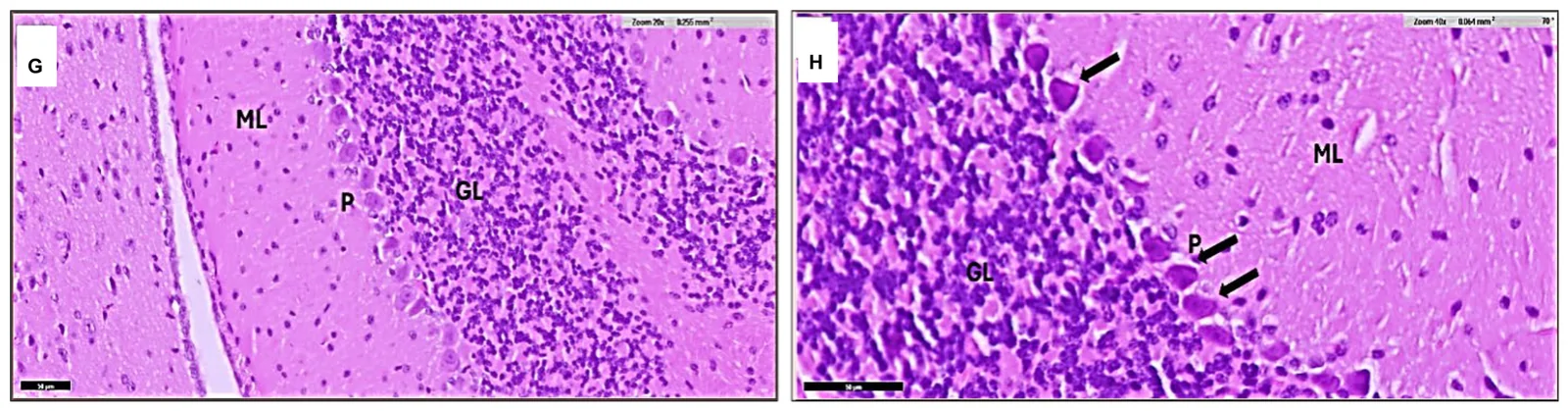
Photomicrographs of H&E-stained cerebellar sections of the control group (**A**,**B**), VPA group (**C**–**E**), BME group (**F**) and BME + VPA group (**G**,**H**). Molecular layer: ML, Granular layer: GL, Purkinje cells: P, Blood vessels: DV, Vacuolations: V.

The cerebellum of the control group exhibited normal histoarchitecture, characterized by an intact molecular layer, regularly aligned Purkinje cells with clear nuclei, and a densely packed granular layer of small, well-preserved cells. Overall laminar organization was maintained without any degenerative changes (Figure 10A,B).

The cerebellar cortex of the VPA group exhibited marked structural disorganization, characterized by disruption of the molecular layer, loss of Purkinje cells, and an apparent increase in granular cell layer density (Figure 10C). Additional sections revealed pronounced loss of the Purkinje cell layer, abnormal morphology of the remaining cells, dilated blood vessels, and poor delineation between the molecular and granular layers (Figure 10D). Moreover, severe disruption of the molecular layer with numerous vacuolations, irregular granular layer distribution, dilated blood vessels, and abnormally shaped Purkinje cells was observed (Figure 10E).

Sections from the BME group exhibited a normal cerebellar architecture, characterized by a well-organized molecular layer, intact Purkinje cells with polygonal to rounded morphology and clear nuclei, and a densely packed granular layer (Figure 10F).

The cerebellum of the BME + VPA group exhibited preserved histoarchitecture, characterized by distinct and regularly arranged Purkinje cells, a well-organized molecular layer, and a densely packed granular layer composed of small, rounded cells without evident degenerative changes (Figure 10G). Additionally, the molecular layer showed a normal arrangement, with Purkinje cells displaying large polygonal morphology and rounded, clear nuclei, while the granular layer remained compact and well organized, maintaining structural integrity without pathological alterations (Figure 10H).

## 3. Discussion

The prevalence of autism spectrum disorder (ASD) has risen over time [25]. Despite extensive research, no universally accepted diagnostic criteria or curative therapy exist, and the underlying etiology of ASD remains unclear. Effective management requires a multifaceted strategy incorporating behavioral, educational, and supportive interventions due to the disorder’s complex nature. Current pharmacological treatments primarily target associated symptoms rather than the core features of ASD [26]. Consequently, there is growing interest in exploring natural, plant-derived compounds as adjunct therapeutic options. Such approaches are particularly appealing given the limitations of conventional pharmaceuticals, which often focus on behavioral manifestations while neglecting underlying pathophysiology and frequently causing adverse effects [27].

This study investigated the potential protective effects of *Bacopa monnieri* (L.) Pennell extract (BME) against ASD-like symptoms induced by prenatal exposure to valproic acid (VPA). A comprehensive battery of behavioral tests was employed to evaluate BME as a natural preventive intervention for ASD-related deficits. Our findings demonstrated significant behavioral improvements, accompanied by biochemical evidence of reduced oxidative stress, reflected by decreased MDA levels and restored GSH levels, as well as histological improvements.

Prenatal VPA exposure in this study caused notable developmental impairments, including reduced body weight and size, delayed eye opening, and prolonged latency in the righting reflex test. These observations are consistent with previous reports of growth retardation and developmental delays in VPA-induced autism models [28,29]. Importantly, administration of BME partially restored body weight, improved eye-opening scores, and reduced righting reflex latency, indicating a protective effect on early neurodevelopment. These results are in line with earlier studies demonstrating the neuroprotective and developmental-supportive properties of *Bacopa monnieri* in both human and animal models [28,29].

The negative geotaxis test, which evaluates motor coordination, revealed significant motor deficits in pups prenatally exposed to VPA, consistent with previous findings in VPA models [30]. Treatment with BME markedly improved performance in this test, suggesting a protective effect against vestibular and postural dysfunctions. These results align with reports that the antioxidant and neuromodulatory properties of *Bacopa monnieri* enhance motor control and coordination [31].

In the open field test, VPA-exposed pups exhibited pronounced hyperactivity and abnormal exploratory behavior, corroborating earlier studies linking VPA exposure to increased locomotor activity [32]. Particularly, BME treatment improved locomotor balance and significantly reduced hyperactivity, consistent with clinical evidence showing that *Bacopa monnieri* supplementation decreases hyperactivity and enhances attention in patients with ADHD [33].

Concerning sensory function, the hot plate test demonstrated that VPA-exposed mice exhibited heightened nociceptive sensitivity, reflecting sensory processing abnormalities characteristic of autism models [34]. Administration of BME significantly increased the pain threshold, indicating its ability to modulate nociceptive responses and restore sensory balance, consistent with previous reports of the analgesic and neuroprotective effects of *Bacopa monnieri* [35].

Furthermore, VPA exposure induced repetitive and stereotypical behaviors, including increased marble burying, prolonged grooming, elevated rearing events, and decreased spontaneous alternation in the Y-maze. These behaviors resemble compulsive-like traits described in ASD models [36]. BME treatment significantly attenuated these repetitive behaviors, supporting its modulatory effects on synaptic proteins and neurotransmitter systems to alleviate aberrant behaviors [33].

VPA-exposed mice exhibited reduced sociability in the three-chamber test, reflecting social deficits consistent with previous reports linking prenatal VPA exposure to impaired social behavior [36,37]. Notably, BME treatment improved social preference and partially restored social interaction, supporting evidence that *Bacopa monnieri* enhances social and cognitive behaviors in both clinical and pediatric populations [38,39]. Collectively, these findings highlight the broad neuroprotective and behavioral-modulating potential of BME in mitigating VPA-induced ASD-like deficits.

Biochemical analysis revealed that co-administration of BME markedly reduced VPA-induced lipid peroxidation, lowering MDA levels by approximately 27% compared with the VPA-only group. Although MDA levels did not fully return to control values, this partial recovery aligns with previous findings indicating that antioxidant treatments often reduce oxidative stress markers toward baseline levels, although not necessarily to control values [40]. These findings support the notion that BME alleviates, but does not eliminate, VPA-induced oxidative damage. Similarly, BME co-treatment significantly attenuated VPA-induced GSH depletion compared with the VPA-only group. Although GSH levels did not fully return to control values, this partial restoration is biologically meaningful, given the central role of GSH as the principal intracellular antioxidant in neutralizing reactive oxygen species. This pattern is consistent with previous reports showing that antioxidant co-treatment produces significant, although incomplete, recovery of GSH levels in VPA-induced oxidative stress models [41] and in other oxidative stress conditions, where complete normalization to control levels is not considered a prerequisite for demonstrating meaningful antioxidant efficacy. The hippocampus is critically involved in learning, memory, and emotional regulation and is highly vulnerable to pathological insults such as oxidative stress, neuroinflammation, and neurotransmitter imbalance [42]. In the present study, VPA administration induced pronounced histopathological alterations in the dentate gyrus, including disorganization and reduced density of the granular cell layer, irregular granule cells with pyknotic nuclei, extensive vacuolations, and markedly dilated blood vessels. Additionally, pyramidal neurons exhibited abnormal morphology, reflecting severe disruption of hippocampal cytoarchitecture. These observations are consistent with previous reports demonstrating that prenatal VPA exposure results in neuronal degeneration and impaired hippocampal organization [43,44].

Conversely, treatment with BME markedly attenuated VPA-induced hippocampal damage. The granular cell layer appeared compact and well organized, with rounded granule cells exhibiting large, deeply stained nuclei. Moreover, pyramidal neurons showed partial restoration toward normal morphology, accompanied by preserved vascular integrity. These histological improvements indicate a protective role of BME in maintaining hippocampal structural integrity. In addition to the antioxidant effects demonstrated in the present study, previous studies have suggested that the neuroprotective effects of *Bacopa monnieri* may also be attributed to its anti-inflammatory properties [39]. These findings are in agreement with earlier studies demonstrating that *Bacopa monnieri* preserves hippocampal architecture in various experimental models, including aluminum-induced neurotoxicity, as evidenced by ultrastructural analysis [45], and cold stress-induced neurodegeneration, confirmed by histomorphometric assessments [46].

The cerebellum is essential for motor coordination, balance, cognitive processing, and the integration of sensory input with motor output [47]. In the present study, VPA administration induced pronounced cerebellar histopathological alterations, including marked degeneration of Purkinje cells, disruption of the normal organization of the granular layer, and a reduced density of granule cells. These findings are consistent with previous reports demonstrating that VPA induces cerebellar damage, particularly affecting Purkinje cell morphology and granule cell arrangement [48].

In contrast, BME treatment markedly mitigated these VPA-induced cerebellar alterations. Purkinje cells appeared more regularly arranged with reduced degenerative changes, and the granular layer maintained a relatively intact architecture. These observations underscore the neuroprotective effect of BME in preserving cerebellar structural integrity, which is likely attributable to its antioxidant properties, as previously reported in experimental models of cerebellar injury [49].

## 4. Materials and Methods

### 4.1. Animals and Ethical Compliance

This study was approved by the Biomedical Ethics Research Committee of King Abdulaziz University (No. 238-25) and conducted in accordance with the Animal Care and Use Committee guidelines of King Fahad Medical Research Center (Protocol No. ACUC-24-08-06). A total of 96 C57BL/6 mice (male and female, 8–10 weeks, 17–22 g) were utilized in this study. To generate the prenatal autism model, mice were housed overnight for mating, and the presence of a vaginal plug the following morning was recorded as embryonic day 0 (E0) [50]. Pregnant females were individually housed until parturition. After weaning, offspring were kept in groups of five in translucent acrylic cages with regularly changed dust-free bedding, maintained at 25 ± 1 °C under a reversed 12-h light/dark cycle, with ad libitum access to food and water.

### 4.2. Bacopa monnieri Extract (BME) Preparation

Commercially available *Bacopa monnieri* (BM) supplement capsules were purchased from iHerb (USA). The contents of the capsules were removed and finely powdered to ensure uniformity. Immediately before administration, the powder was freshly suspended in distilled water to achieve the required dose of 400 mg/kg body weight. The suspension was vortex-mixed thoroughly to obtain a homogeneous preparation and was prepared fresh each day prior to use. The selected dose was based on a previous preclinical study demonstrating the safety and efficacy of 400 mg/kg *Bacopa monnieri* in mice [51,52,53]. The prepared suspension was administered orally once daily to pregnant mice throughout the gestational period using a sterile oral gavage needle.

#### The Chemical Characterization of the Preparation of *Bacopa monnieri* Extract

The dried *Bacopa monnieri* plant material was finely ground, sieved, and an accurately weighed 0.6 g portion was extracted with 6 mL methanol in a 16 mL stoppered glass tube. The mixture was vortex-mixed for 1 min, sonicated for 10 min, and centrifuged at 5000 rpm for 10 min. The resulting supernatant was collected and analyzed by HPLC-DAD (10 μL injection; detection at 330 and 360 nm) for quantitative analysis and by LC-ESI-Ion Trap MS (5 μL injection; positive ion mode) for compound identification.

For the commercial *Bacopa monnieri* tablets, two tablets were finely powdered, and a 2.0 g portion was extracted with 10 mL methanol by vortex mixing for 2 min, followed by sonication for 15 min and centrifugation at 5200 rpm for 10 min. The supernatant was filtered through a 0.22 μm PTFE membrane filter, concentrated under a gentle nitrogen stream, and analyzed by LC-ESI-Ion Trap MS (5 μL injection; positive ion mode).

Detailed chemical characterization of the *Bacopa monnieri* extract is provided in the Supplementary Materials, including File S1: Characterization of the Chemical Constituents of *Bacopa monnieri* and File S2: Characterization of the Major Constituents of *Bacopa monnieri*.

### 4.3. Induction of Autism

Pregnant C57BL/6 females received a single intraperitoneal injection of valproic acid (VPA) (600 mg/kg; Sigma-Aldrich, P4543, St. Louis, MO, USA) dissolved in 0.9% saline on embryonic day 12 (E12), following the protocol of Kim et al. [50].

### 4.4. Experimental Design

Ninety-six C57BL/6 mice (male and female, 8–10 weeks, 17–22 g) were housed overnight for mating, and the presence of a vaginal plug the following morning was recorded as embryonic day 0 (E0). Pregnant females were randomly allocated into four groups (*n* = 12): Control group: pregnant mice were administered intraperitoneally with saline solution on E12. VPA group: pregnant mice were injected with valproic acid (600 mg/kg, i.p.) on E12. BME group: mice were administered with *B. monnieri* extract (400 mg/kg, oral) daily throughout pregnancy period. BME + VPA group: mice were administered *B. monnieri* extract (400 mg/kg, oral) daily throughout pregnancy plus valproic acid (600 mg/kg, i.p.) on E12.

**Figure 11:**
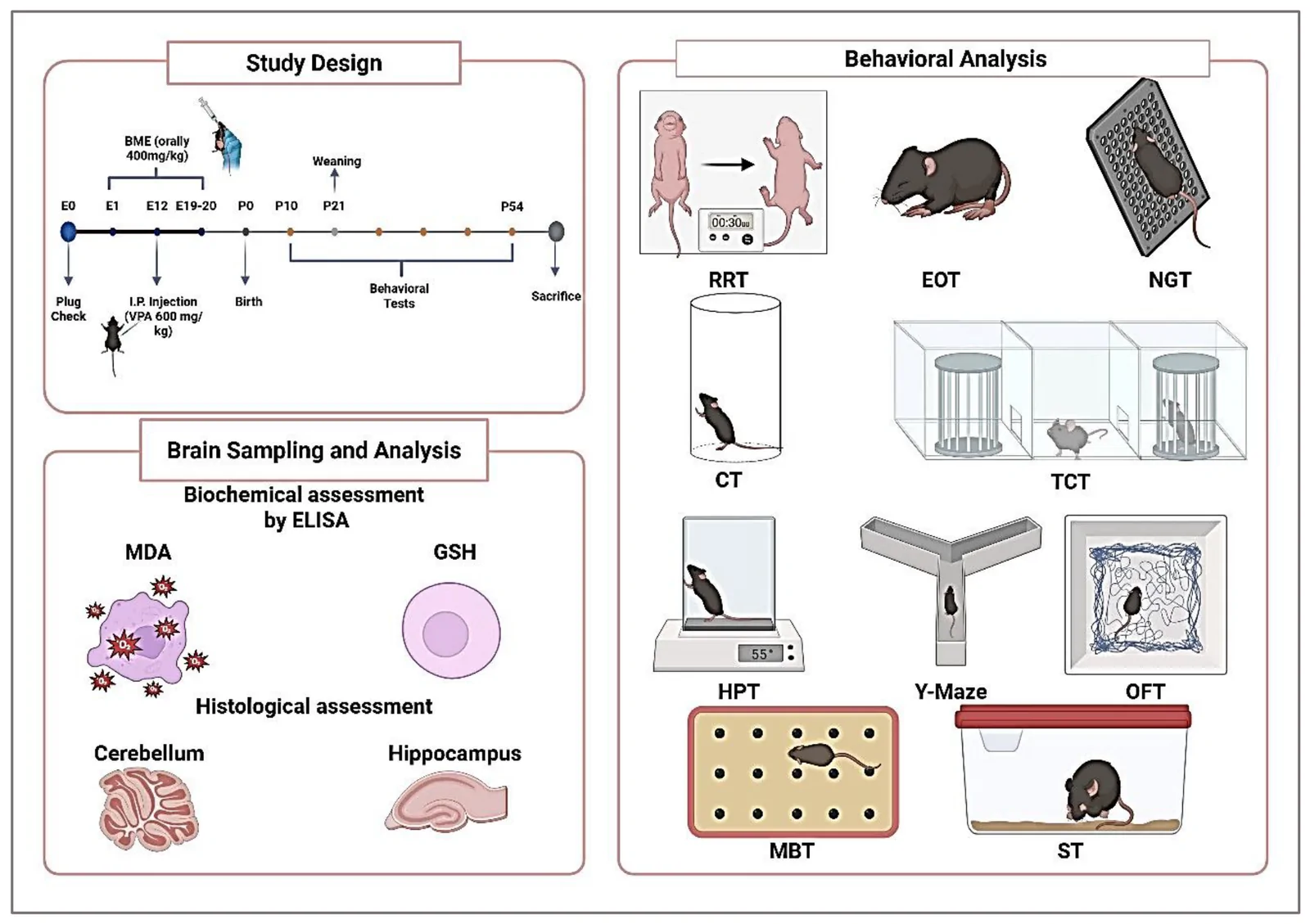
Schematic overview of the study design and experimental assessments, including behavioral tests (RRT, EOT, NGT, CT, TCT, HPT, Y-maze, OFT, MBT, ST), biochemical analysis of oxidative stress markers (MDA and GSH) by ELISA, and histological evaluation of the hippocampus and cerebellum.

A comprehensive assessment was performed, including behavioral (RRT, EOT, NGT, CT, TCT, HPT, Y-Maze, OFT, MBT, ST), biochemical (MDA and GSH by ELISA), and histological analyses of the hippocampus and cerebellum. These evaluations provided an integrated profile of behavioral performance, oxidative stress, and neuropathological alterations associated with ASD (Figure 11).

### 4.5. Behavioral Tests

#### 4.5.1. Assessment of Developmental Abnormalities

##### Eyes Opening Test (EOT)

Eye opening was evaluated daily from postnatal day P14 to P18 and scored as follows: 0 = both eyes closed, 1 = one eye open, and 2 = both eyes open [54].

##### Righting Reflex Test (RRT)

The righting reflex was assessed from postnatal day P10 to P13, following Soria-Ortiz et al. [55]. Each pup was placed supine on a flat surface for 5 sec, then released, and the time required to return to a prone position was recorded.

#### 4.5.2. Assessment of Motor Deficits

##### Negative Geotaxis Test (NGT)

Motor coordination was assessed by placing each pup facing downward on a 45° incline. The time required to turn 180° and climb upward was recorded, following Wang et al. [56]. Pups that failed to climb within 30 s were assigned the maximum time.

#### 4.5.3. Assessment of Social Cognitive Dysfunction

##### Three-Chamber Test (TCT)

Sociability was assessed using a three-chamber apparatus (60 × 40 × 20 cm) with openings allowing free movement. Following a 10-min habituation, the test was conducted for 10 min. A novel “stranger” mouse was placed in one chamber, while the remaining chambers were empty. Time spent in each chamber was recorded, and the social preference index was calculated as:

Social Preference Index (%) = [Time in stranger chamber/(Time in empty chambers + Time in stranger chamber)] × 100

#### 4.5.4. Assessment of Repetitive Behavior

##### Y-Maze Test (YMT)

The Y-maze spontaneous alternation test was used to assess repetitive behavior in mice [57]. The apparatus consists of three arms (A, B, and C), each 10 cm wide and 15 cm high. Mice were placed in the same starting arm and allowed to explore for 5 min while video-recorded from above. The maze was cleaned with 70% ethanol between trials. Spontaneous alternation was calculated as: Spontaneous alternation (%) = [Total alternations/(Total arm entries − 2)] × 10 [58].

##### Cylinder Test (CT)

In the cylinder test, each mouse was placed in a transparent cylindrical chamber for 3 min. Repetitive behavior was assessed by counting the number of forelimb wall contacts during rearing [59].

##### Marble Burying Test (MBT)

In the marble burying test, 20 marbles were placed on the surface of clean bedding. The number of marbles buried during a 30-min session was recorded by investigators blinded to treatment. This test assesses repetitive, compulsive-like behaviors in mice [60].

##### Splash Test (ST)

Each mouse was placed in an individual cage and sprayed on the dorsal coat with a 10% sucrose solution. Grooming time was then recorded for 5 min [61].

#### 4.5.5. Assessment of Hyperactivity

##### Open Field Test (OFT)

The open field test was performed as previously described [62] to assess hyperactivity at P35. Mice were placed in the center of a 45 × 45 × 34 cm open-field arena and allowed to explore for 5 min. The arena was cleaned with 70% ethanol between trials. Video recordings were analyzed using EthoVision XT (version 15.0; Noldus, Wageningen, The Netherlands) to measure total distance traveled.

#### 4.5.6. Assessment of Sensory Abnormalities

##### Hot Plate Test (HPT)

The hot plate test was used to assess nociceptive responses. Mice were placed in an open-ended cylindrical chamber on a hot plate maintained at 55 ± 1 °C. Paw licking and jumping were recorded as nociceptive behaviors [63]. A 30-s cut-off was used to prevent tissue damage, and this value was considered the reaction time when reached [64].

### 4.6. Brain Sample Collection

At the end of the study, mice were anesthetized with isoflurane, and blood samples were collected. Animals were then euthanized by cervical dislocation, and brains were immediately harvested. Brains designated for histopathology were fixed in 10% formalin and processed after 48 h, while remaining samples were stored at −80 °C for biochemical analysis.

### 4.7. Histological Examination

Histological processing was performed as described by Alqurashi et al. [65]. Brains were sectioned into coronal and sagittal slices, placed in histocassettes, and processed for 15 h using a tissue processor (Tissue Tek^®^ VIP™ 5 Jr-E2, Sakura, Japan). Tissues were embedded in paraffin, trimmed, and cut into 3 µm sections using a microtome (LEICA RM 2125 RTS). Sections were mounted on slides and stained with hematoxylin and eosin (H&E) using an automated stainer (Bio-Optica, Milan, Italy). The hippocampus and cerebellum were examined at 5×, 10×, 20×, and 40× magnifications and imaged using a Philips IntelliSite Pathology scanner (Amsterdam, The Netherlands).

### 4.8. Biochemical Studies

#### Determination of MDA and GSH by ELISA

The levels of malondialdehyde (MDA) and reduced glutathione (GSH) were determined in brain tissue using commercially available competitive ELISA kits (MDA: SEKSM-0058; GSH: SEKSM-0053; Solarbio, Beijing, China) according to the manufacturer’s instructions. Absorbance was measured at 450 nm using a microplate reader. The concentrations of MDA and GSH were calculated by comparing the absorbance values of the samples with calibration curves generated from standard solutions of known concentrations. MDA levels are expressed as ng/mL, whereas GSH levels are expressed as µg/mL.

### 4.9. Statistical Analysis

Statistical analyses were performed using GraphPad Prism (v.10, GraphPad Software, San Diego, CA, USA). Differences between groups were assessed by one-way or two-way ANOVA followed by Tukey’s multiple comparison test. Data are expressed as mean ± SEM, with sample sizes indicated in the figure legends. *p* < 0.05 in the obtained data was considered statistically significant.

## 5. Conclusions

Prenatal exposure to valproic acid (VPA) successfully induced autism spectrum disorder (ASD)-like phenotypes in mice, manifested by impaired social interaction, increased repetitive and stereotypic behaviors, hyperactivity, and motor dysfunction. These behavioral deficits were associated with pronounced biochemical disturbances, notably oxidative imbalance, as indicated by elevated malondialdehyde (MDA) levels and reduced glutathione (GSH) content, underscoring the role of oxidative stress in ASD pathophysiology. Moreover, VPA exposure elicited significant histopathological alterations in the hippocampus and cerebellum, including neuronal degeneration, Purkinje cell loss, and disrupted tissue architecture.

Treatment with *Bacopa monnieri* (BM) extract markedly attenuated VPA-induced behavioral abnormalities, including hyperactivity, repetitive behaviors, and social deficits. BM administration also mitigated oxidative stress by reducing MDA levels and restoring GSH concentrations, thereby enhancing antioxidant defenses. Additionally, BM exerted a neuroprotective effect by preserving hippocampal and cerebellar cytoarchitecture and limiting neuronal degeneration. So, these findings highlight the therapeutic potential of *Bacopa monnieri* as a natural preventive and neuroprotective strategy against ASD-like manifestations.

## Figures and Tables

**Table 1:** Characterized major constituents of *Bacopa monnieri* extract.

Rt, Min	Compound (s)	M Wt	[M + H]+, *m*/*z*
14.12	Cucurbitacin I	514	497
17.78	Cucurbitacin I	514	497
26.06	Cucurbitacin I	514	497
19.21	Senegenin	536	473
13.6 to 21.2	Bacoside	Varied	455
14.3 to 17	Esculentic acid	488	489
10.52	Luteolin 7-glucuronide	462	463
26.8	Bacoside A	766	749.4
29.4	(9Z,12E)-15,16-Dihydroxyoctadeca-9,12-dienoic acid	312	277
16	Luteolin	286	287
11.9	Genistein-4′-glucuronide	446	447
8.8	6′-(p-Hydroxybenzoyl)mussaenosidic acid	496	283
9.32	Echinacoside	786	325

**Table 2:** Effect of BME on MDA content in brain of mice exposed to VPA.

Groups	MDA Content (ng/mL)	*p*-Value	
Control	3.82 ± 0.24	Control vs. VPA (**)	0.0014
VPA	6.45 ± 0.31	Control vs. BME (ns)	0.9997
BME	3.98 ± 0.22	Control vs. BME + VPA (ns)	0.5989
BME + VPA	4.72 ± 0.26	VPA vs. BME + VPA (*)	0.0247

Values are expressed as mean ± SEM (n = 6 per group). Statistical analysis was performed using one-way ANOVA followed by Tukey’s post hoc test. Significant differences are indicated as * *p* < 0.05 and ** *p* < 0.01, ns: non-significant.

**Table 3:** Effect of BME on GSH content in brain of mice exposed to VPA.

Groups	GSH Content (µg/mL)	*p*-Value	
Control	24.56 ± 3.32	Control vs. VPA (**)	0.0032
VPA	13.36 ± 2.21	Control vs. BME (ns)	0.7526
BME	23.52 ± 4.14	Control vs. BME + VPA (ns)	0.4668
BME + VPA	21.81 ± 3.71	VPA vs. BME + VPA (*)	0.0342

Values are expressed as mean ± SEM (n = 6 per group). Statistical analysis was performed using one-way ANOVA followed by Tukey’s post hoc test. Significant differences are indicated as * *p* < 0.05 and ** *p* < 0.01, ns: non-significant.

## Data Availability

Data supporting the findings of this study are available from the corresponding authors upon reasonable request.

## Supplementary Materials

The following supporting information can be downloaded at https://www.mdpi.com/article/10.3390/molecules31162783/s1, File S1: Characterization of the Chemical Constituents of *Bacopa monnieri;* File S2: Characterization of the Major Constituents of *Bacopa monnieri*.

## Abbreviations

ASD	Autism Spectrum Disorder
BM	*Bacopa monnieri*
BME	*Bacopa monnieri* Extract
VPA	Valproic Acid
E12	Embryonic Day 12
MDA	Malondialdehyde
GSH	Glutathione
HDAC	Histone Deacetylase
ROS	Reactive Oxygen Species

## References

[1] American Psychiatric Association (2013). Diagnostic and Statistical Manual of Mental Disorders.

[2] Zeidan J., Fombonne E., Scorah J., Ibrahim A., Durkin M.S., Saxena S., Yusuf A., Shih A., Elsabbagh M. (2022). Global prevalence of autism: A systematic review update. Autism Res..

[3] Modabbernia A., Velthorst E., Reichenberg A. (2017). Environmental risk factors for autism: An evidence-based review of systematic reviews and meta-analyses. Mol. Autism.

[4] Sandin S., Lichtenstein P., Kuja-Halkola R., Larsson H., Hultman C.M., Reichenberg A. (2014). The familial risk of autism. JAMA.

[5] Frustaci A., Neri M., Cesario A., Adams J.B., Domenici E., Dalla Bernardina B., Bonassi S. (2012). Oxidative stress-related biomarkers in autism: Systematic review and meta-analyses. Free Radic. Biol. Med..

[6] Pangrazzi L., Balasco L., Bozzi Y. (2020). Oxidative stress and immune system dysfunction in autism spectrum disorders. Int. J. Mol. Sci..

[7] Robertson C.E., Ratai E.M., Kanwisher N. (2016). Reduced GABAergic action in the autistic brain. Curr. Biol..

[8] Lee E., Lee J., Kim E. (2017). Excitation/inhibition imbalance in animal models of autism spectrum disorders. Biol. Psychiatry.

[9] Chauhan A., Chauhan V. (2006). Oxidative stress in autism. Pathophysiology.

[10] Rossignol D.A., Frye R.E. (2014). Evidence linking oxidative stress, mitochondrial dysfunction, and inflammation in the brain of individuals with autism. Front. Physiol..

[11] Botelho R.M., Silva A.L.M., Borbély A.U. (2024). The autism spectrum disorder and its possible origins in pregnancy. Int. J. Environ. Res. Public Health.

[12] Mabunga D.F.N., Gonzales E.L.T., Kim J., Kim K.C., Shin C.Y. (2015). Exploring the validity of the valproic acid animal model of autism. Exp. Neurobiol..

[13] McCracken J.T., McGough J., Shah B., Cronin M., Hong D., Aman M.G., Arnold L.E., Lindsay R., Nash P., Hollway J. (2002). Risperidone in children with autism and serious behavioral problems. N. Engl. J. Med..

[14] Owen R., Sikich L., Marcus R.N., Corey-Lisle P.K., Manos G., McQuade R.D., Findling R.L. (2009). Aripiprazole in the treatment of irritability in children and adolescents with autistic disorder. Pediatrics.

[15] Posey D.J., Erickson C.A., McDougle C.J. (2008). Developing drugs for core social and communication impairment in autism. Child Adolesc. Psychiatr. Clin. N. Am..

[16] Williams K., Brignell A., Randall M., Silove N., Hazell P. (2010). Selective serotonin reuptake inhibitors (SSRIs) for autism spectrum disorders (ASD). Cochrane Database Syst. Rev..

[17] Lamy M., Erickson C.A. (2018). Pharmacological management of autism spectrum disorder: Current status and future directions. CNS Drugs.

[18] Bhandari R., Kuhad A. (2018). Neuropsychopharmacotherapeutic efficacy of natural products in animal models of autism. Eur. J. Pharmacol..

[19] Rossignol D.A., Frye R.E. (2012). A review of research trends in physiological abnormalities in autism spectrum disorders: Immune dysregulation, inflammation, oxidative stress, mitochondrial dysfunction and environmental toxicant exposures. Mol. Psychiatry.

[20] Bjørklund G., Meguid N.A., El-Bana M.A., Tinkov A.A., Dadar M. (2020). Oxidative stress in autism spectrum disorder. Mol. Neurobiol..

[21] Kumar A., Sharma S. (2021). Plant-derived bioactive compounds in autism: Insights into therapeutic mechanisms. Front. Pharmacol..

[22] Neto L.J.V., Reverete M., Junior R.C.M., Machado N.M., Joshi R.K., dos Santos Buglio D., Lamas C.B., Direito R., Laurindo L.F., Tanaka M. (2024). The neuroprotective and cognitive-enhancing effects of *Bacopa monnieri*: A systematic review. Preprints.

[23] Banerjee S., Anand U., Ghosh S., Ray D., Ray P., Nandy S., Deshmukh G.D., Tripathi V., Dey A. (2021). Bacosides from *Bacopa monnieri* extract: An overview of the effects on neurological disorders. Phytother. Res..

[24] Calabrese C., Gregory W.L., Leo M., Kraemer D., Bone K., Oken B. (2008). Effects of a standardized *Bacopa monnieri* extract on cognitive performance, anxiety, and depression in the elderly: A randomized, double-blind, placebo-controlled trial. J. Altern. Complement. Med..

[25] Shaw K.A., Williams S., Patrick M.E., Valencia-Prado M., Durkin M.S., Howerton E.M., Ladd-Acost C.M., Pas E.T., Bakian A.V., Bartholomew P. (2025). Prevalence and early identification of autism spectrum disorder among children aged 4 and 8 years—Autism and Developmental Disabilities Monitoring Network, 16 sites, United States, 2022. MMWR Surveill. Summ..

[26] Manter M.A., Birtwell K.B., Bath J., Friedman N.D.B., Keary C.J., Neumeyer A.M., Palumbo M.L., Thom R.P., Stonestreet E., Brooks H. (2025). Pharmacological treatment in autism: A proposal for guidelines on common co-occurring psychiatric symptoms. BMC Med..

[27] Sachdeva P., Mehdi I., Kaith R., Ahmad F., Anwar M.S. (2022). Potential natural products for the management of autism spectrum disorder. iBrain.

[28] Limpeanchob N., Jaipan S., Rattanakaruna S., Phrompittayarat W., Ingkaninan K. (2008). Neuroprotective effect of *Bacopa monnieri* on beta-amyloid-induced cell death in primary cortical culture. J. Ethnopharmacol..

[29] Naik J.S., Patil R., Somashekara S.R., Ramesha T.J., Suresh T. (2025). Effect of dietary supplementation of Brahmi (*Bacopa monnieri*) on growth, survival and gut histology of Nile tilapia (*Oreochromis niloticus*). J. Adv. Biol. Biotechnol..

[30] Ruhela R.K., Soni S., Sarma P., Prakash A., Medhi B. (2019). Negative geotaxis: An early age behavioral hallmark to VPA rat model of autism. Ann. Neurosci..

[31] Vishnupriya P., Padma V. (2017). A review on the antioxidant and therapeutic potential of *Bacopa monnieri*. React. Oxyg. Species.

[32] Schneider T., Przewłocki R. (2005). Behavioral alterations in rats prenatally exposed to valproic acid: An animal model of autism. Neuropsychopharmacology.

[33] Amin R., Hassan M.S., Mohd Saleh R., Zamri E.N., Tan S.E.O., Amin R.M. (2025). Synergistic effects of multiple bioactive ingredients in the treatment of ADHD in children: A pathophysiological and clinical systematic review. South East Eur. J. Public Health.

[34] Roullet F.I., Lai J.K.Y., Foster J.A. (2013). In utero exposure to valproic acid and autism: A current review of clinical and animal studies. Neurotoxicol. Teratol..

[35] Fatima U., Stough C., Okkay U., Maurya D., Pereira C., Roy S., Ahmad S., Ali S., Elkady W.M., Khan I. (2022). Pharmacological attributes of *Bacopa monnieri* extract: Current updates and clinical manifestation. Front. Nutr..

[36] Nicolini C., Fahnestock M. (2018). The valproic acid-induced rodent model of autism. Exp. Neurol..

[37] Zarate-Lopez D., Torres-Chávez A.L., Gálvez-Contreras A.Y., Gonzalez-Perez O. (2023). Three decades of valproate: A current model for studying autism spectrum disorder. Curr. Neuropharmacol..

[38] Bhardwaj S., Rathee V.S., Rathee N.K. (2023). Efficacy of *Bacopa monnieri* in enhancing cognitive outcomes and its therapeutic potential for ADHD: A metasynthesis. J. Adv. Anal. Healthc. Manag..

[39] Valotto Neto L.J., Reverete de Araujo M., Moretti Junior R.C., Mendes Machado N., Joshi R.K., dos Santos Buglio D., Barbalho Lamas C., Direito R., Fornari Laurindo L., Tanaka M. (2024). Investigating the neuroprotective and cognitive-enhancing effects of Bacopa monnieri: A systematic review focused on inflammation, oxidative stress, mitochondrial dysfunction, and apoptosis. Antioxidants.

[40] Waidande S.S., Kshirsagar M., Thorat V.M., Tiwari D.D., Waidande S., Kshirsagar M.S. (2025). Role of antioxidant supplementation in enhancing chelation therapy for lead-induced oxidative stress in rats. Cureus.

[41] Ahangar N., Naderi M., Noroozi A., Ghasemi M., Zamani E., Shaki F. (2017). Zinc deficiency and oxidative stress involved in valproic acid induced hepatotoxicity: Protection by zinc and selenium supplementation. Biol. Trace Elem. Res..

[42] Banker S.M., Gu X., Schiller D., Foss-Feig J.H. (2021). Hippocampal contributions to social and cognitive deficits in autism spectrum disorder. Trends Neurosci..

[43] Luhach K., Kulkarni G.T., Singh V.P., Sharma B. (2021). Vinpocetine amended prenatal valproic acid-induced features of ASD possibly by altering markers of neuronal function, inflammation, and oxidative stress. Autism Res..

[44] Saadat M., Taherian A.A., Aldaghi M.R., Raise-Abdullahi P., Sameni H.R., Vafaei A.A. (2023). *Prangos ferulacea* ameliorates behavioral alterations, hippocampal oxidative stress markers, and apoptotic deficits in a rat model of autism induced by valproic acid. Brain Behav..

[45] Nannepaga J.S., Korivi M., Tirumanyam M., Bommavaram M., Kuo C.H. (2014). Neuroprotective effects of *Bacopa monniera* whole-plant extract against aluminum-induced hippocampus damage in rats. Chin. J. Physiol..

[46] Saravana Kumar S., Saraswathi P., Vijayaraghavan R. (2015). Effect of *Bacopa monniera* on cold stress induced neurodegeneration in hippocampus of Wistar rats: A histomorphometric study. J. Clin. Diagn. Res..

[47] Biswas D., Sontag E.D., Cowan N.J. (2025). An exact active sensing strategy for a class of bio-inspired systems. Eur. J. Control.

[48] Eid L.T.M., El-Habeby M.M., Issa N.M., El-Dien N.M.N. (2023). Evaluation of prenatal administration of valproic acid on the cerebellum of albino rat offspring. Int. J. Health Sci..

[49] Shahid M., Subhan F., Ali G., Ullah I., Alam J., Ullah S., Rauf K. (2017). Neuroprotective effect of *Bacopa monnieri* against morphine-induced histopathological changes in the cerebellum of rats. Pak. J. Pharm. Sci..

[50] Kim U.J., Hong N., Ahn J.C. (2022). Photobiomodulation attenuated cognitive dysfunction and neuroinflammation in a prenatal valproic acid-induced autism spectrum disorder mouse model. Int. J. Mol. Sci..

[51] Karim R., Khan A.F., Yeasmin R., Akter J., Akter T. (2020). An evaluation of hepatoprotective activity of aqueous and ethanolic extracts of *Bacopa monnieri* (L.) against paracetamol-induced hepatotoxicity in Swiss albino mice. Eur. J. Biomed. Pharm. Sci..

[52] Abhishek M., Rubal S., Rohit K., Rupa J., Phulen S., Gurjeet K., Raj S.A., Manisha P., Alka B., Ramprasad P. (2022). Neuroprotective effect of the standardised extract of *Bacopa monnieri* (BacoMind) in valproic acid model of autism spectrum disorder in rats. J. Ethnopharmacol..

[53] Sudershan B., Chowta M.N., Ullal S.D., Rajeshwari S., Kumar Sayeli V., Shivaprasad S., Srivastava P. (2018). Effect of Bacopa Monnieri on Ethanol-induced Anxiolysis and Withdrawal Anxiety in Wistar Rats. Indian J. Physiol. Pharmacol..

[54] Al Sagheer T., Haida O., Balbous A., Francheteau M., Matas E., Fernagut P.O., Jaber M. (2018). Motor impairments correlate with social deficits and restricted neuronal loss in an environmental model of autism. Int. J. Neuropsychopharmacol..

[55] Soria-Ortiz M.B., Reyes-Ortega P., Martínez-Torres A., Reyes-Haro D. (2021). A functional signature in the developing cerebellum: Evidence from a preclinical model of autism. Front. Cell Dev. Biol..

[56] Wang R., Tan J., Guo J., Zheng Y., Han Q., So K.F., Yu J., Zhang L. (2018). Aberrant development and synaptic transmission of cerebellar cortex in a VPA-induced mouse autism model. Front. Cell Neurosci..

[57] Markram K., Rinaldi T., La Mendola D., Sandi C., Markram H. (2008). Abnormal fear conditioning and amygdala processing in an animal model of autism. Neuropsychopharmacology.

[58] Campolongo M., Kazlauskas N., Falasco G., Urrutia L., Salgueiro N., Höcht C., Depino A.M. (2018). Sociability deficits after prenatal exposure to valproic acid are rescued by early social enrichment. Mol. Autism.

[59] Fleming S.M., Ekhator O.R., Ghisays V. (2013). Assessment of sensorimotor function in mouse models of Parkinson’s disease. J. Vis. Exp..

[60] Angoa-Pérez M., Kane M.J., Briggs D.I., Francescutti D.M., Kuhn D.M. (2013). Marble burying and nestlet shredding as tests of repetitive, compulsive-like behaviors in mice. J. Vis. Exp..

[61] Lefter R., Ciobica A., Timofte D., Ababei D., Dobrin R., Luca A., Trifan A., Stanciu C., Sfarti C. (2020). A new biological approach in generating an irritable bowel syndrome rat model—Focusing on depression in sucrose splash test and body weight change. Rom. Biotechnol. Lett..

[62] Kim J.W., Seung H., Kwon K.J., Ko M.J., Lee E.J., Oh H.A., Choi C.S., Kim K.C., Gonzales E.L., You J.S. (2014). Subchronic treatment of donepezil rescues impaired social, hyperactive, and stereotypic behavior in a valproic acid-induced animal model of autism. PLoS ONE.

[63] Ashagrie G., Abebe A., Umer S. (2023). Analgesic and anti-inflammatory activities of 80% methanol extract and solvent fractions of *Ehretia cymosa* Thonn leaves in rodents. J. Exp. Pharmacol..

[64] İnaltekin A., Kıvrak Y. (2021). Evaluation of the effect of vortioxetine on pain threshold by hot-plate test in mice. Noropsikiyatri Ars..

[65] Alqurashi G.K., Hindi E.A., Zayed M.A., Abd El-Aziz G.S., Alturkistani H.A., Ibrahim R.F., Al-Thepyani M.A., Bakhlgi R., Alzahrani N.A., Ashraf G.M. (2022). The impact of chronic unpredictable mild stress-induced depression on spatial, recognition and reference memory tasks in mice: Behavioral and histological study. Behav. Sci..

