# Supplementary material for: Protective Effects of Prenatal Bacopa monnieri Extract Against Valproic Acid-Induced Autism-like Behavioral and Neurohistological Alterations in Mice
Source: Molecules. 2026 Aug 10;31(16):2783. doi: 10.3390/molecules31162783 (PMC13515057; doi:10.3390/molecules31162783)
Supplement: Supplementary file 1 [file molecules-31-02783-s001.zip › molecules-4296639-supplementary/File S1.pdf]

Analysis Report

Contents

1. Report information 2
2. Study Objective 2
3. Sample Information 2
4. Analytical Methodology 2
5. Results & Interpretation 3

kfmrc-tfs@kau.edu.sa
TFSKFMRC

1. Report information

**Laboratory Name**	Toxicology & Forensic Science Unit, King Fahd Medical Research Center
**Client Name**	Doctor Maha Ibrahim
**Request Number**	
**Project Title**	Characterization of chemical constituents of Bacopa monnieri, Brahmi in leaves, and Bacopa Tablets
**Report Date**	Jun 26, 2026
**Instrument Used**	LCQ Fleet – Thermo-Scientific LC-ESI-IT-MS
**Prepared By**	Name: **Prof. Alaa Khedr** Signature
**Approved By**	Name: Signature

2. Study Objective

Characterization of chemical constituents of Bacopa monnieri, Brahmi in leaves and Bacopa Tablets

3. Sample Information

**Number of samples:**	2
**Sample name/ ID:**	Dried leaves of Bacopa monnieri leveas<br>Bacopa Tablets
**Nature (powder/liquid):**	Dried
**Volume/weight:**	Ca 5 g<br>Bottle Tablets
**Sample solvent:**	N/A
**Storage Temperature:**	-20 °C

4. Analytical Methodology

Instrumentation and chromatographic conditions

<u>LC-MS</u>

Analysis was carried out using a Thermo Scientific LTQ-XL linear ion trap mass spectrometer coupled with an Accela autosampler and Accela pump. Electrospray ionization was used as the ion source, and the system was operated with Xcalibur software. The chromatographic column was an Eclipse Plus C18 column (5.0 µm, 4.6 × 150 mm), with column oven temperature maintained at 25 ± 2 °C and tray temperature at 25 °C. The mobile phase consisted of acetonitrile as line A and an aqueous phase containing ammonium acetate trihydrate and formic acid as line B. Gradient elution was performed at a

kfmrc-tfs@kau.edu.sa
TFSKFMRC

flow rate of 550 µL/min. A scan in MS positive mode/auto-fragmentation at 35 V was applied, m/z 100–1400.

Table. Gradient elution

Time, min	%A	%B
**0**	10	90
**10**	10	90
**25**	50	50
**35**	70	30
**45**	80	20
**55**	95	5
**55.1**	10	90

Procedure:
Extraction

The dried plant material of *Bacopa monnieri* was first ground into a fine powder and then sieved to obtain a uniform particle size, thereby improving extraction efficiency and reproducibility. A 0.6 g portion of the sieved powder was accurately weighed and transferred into a 16-mL stoppered glass test tube. The sample was extracted with 6 mL of methanol by vortex mixing for 1 min to ensure complete wetting and dispersion of the plant powder, followed by sonication for 10 min to enhance the release of extractable constituents into the solvent. The mixture was then centrifuged at 5000 rpm for 10 min to separate the solid plant residue from the liquid extract. The clear supernatant was carefully transferred into a clean vial. A 10 µL aliquot was injected into the HPLC-DAD for quantitative analysis at 330 and 360 nm. Also, a 5 µL aliquot of the extract was injected into the LC-ESI-Ion Trap MS system and analyzed in positive ion mode.

Two Bacopa tablets were ground using a porcelain mortar. A 2 g portion of the powdered tablets was transferred into a test tube, mixed with 10 mL of methanol, vortexed for 2 min, sonicated for 15 min, and centrifuged at 5200 rpm for 10 min. The clear supernatant was then filtered through a 0.22 µm PTFE filtration disk and concentrated under a nitrogen stream. Finally, a 5 µL aliquot of the extract was injected into the LC-ESI-Ion Trap MS system and analyzed in positive ion mode.

5. Results

List of identified compounds:

All compounds are shown in the attached Excel file.

kfmrc-tfs@kau.edu.sa
TFSKFMRC

Bacopa monnieri, Brahmi, is marketed as a nootropic with modest, inconsistently demonstrated cognitive benefits, and a safety profile that is generally acceptable but not trivial, especially regarding GI and thyroid effects.
Bacopa monnieri is a creeping, aquatic Ayurvedic herb traditionally used for memory enhancement, anxiety, epilepsy, and insomnia. Modern supplements usually contain standardized extracts rich in bacosides (triterpenoid saponins), considered the main neuroactive constituents.

Characterized major constituents

Rt, min	Compound(s)	M Wt	[M+H]+, m/z
14.12	Cucurbitacin I	514	497
17.78	Cucurbitacin I	514	497
26.06	Cucurbitacin I	514	497
19.21	Senegenin	536	473
13.6 to 21.2	Bacoside	Varied	455
14.3 to 17	Esculentic acid	488	489
10.52	Luteolin 7-glucuronide	462	463
26.8	Bacoside A	766	749.4
29.4	(9Z,12E)-15,16-Dihydroxyoctadeca-9,12-dienoic acid	312	277
16	Luteolin	286	287
11.9	Genistein-4'-glucuronide	446	447
8.8	6'-(p-Hydroxybenzoyl)mussaenosidic acid	496	283
9.32	Echinacoside	786	325

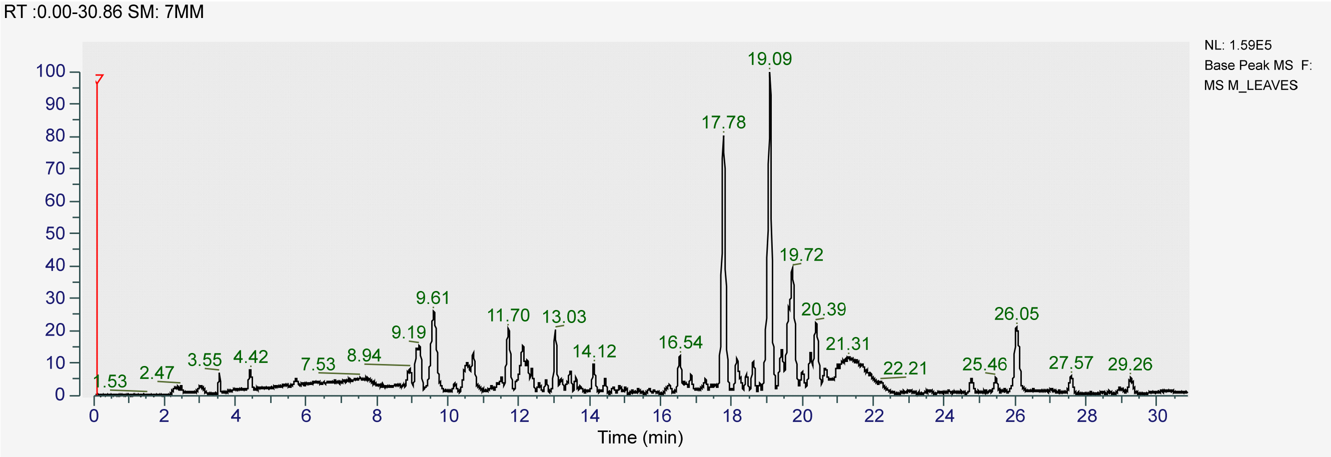

LC-MS TIC chromatogram (m/z 100 – 1400), Leaves

kfmrc-tfs@kau.edu.sa
TFSKFMRC

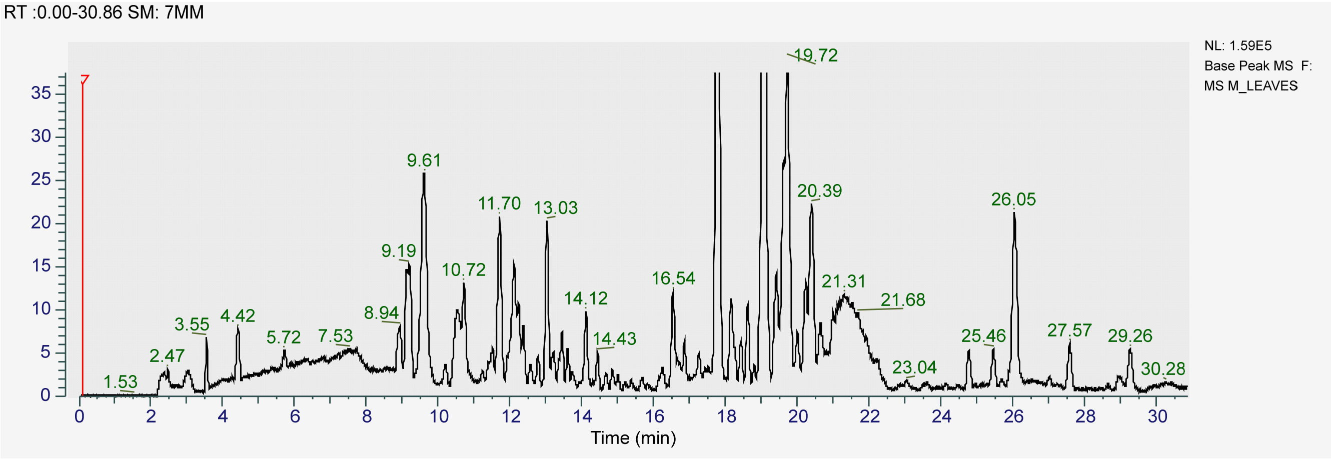

(Zoomed) LC-MS TIC chromatogram (m/z 100 – 1400), leaves

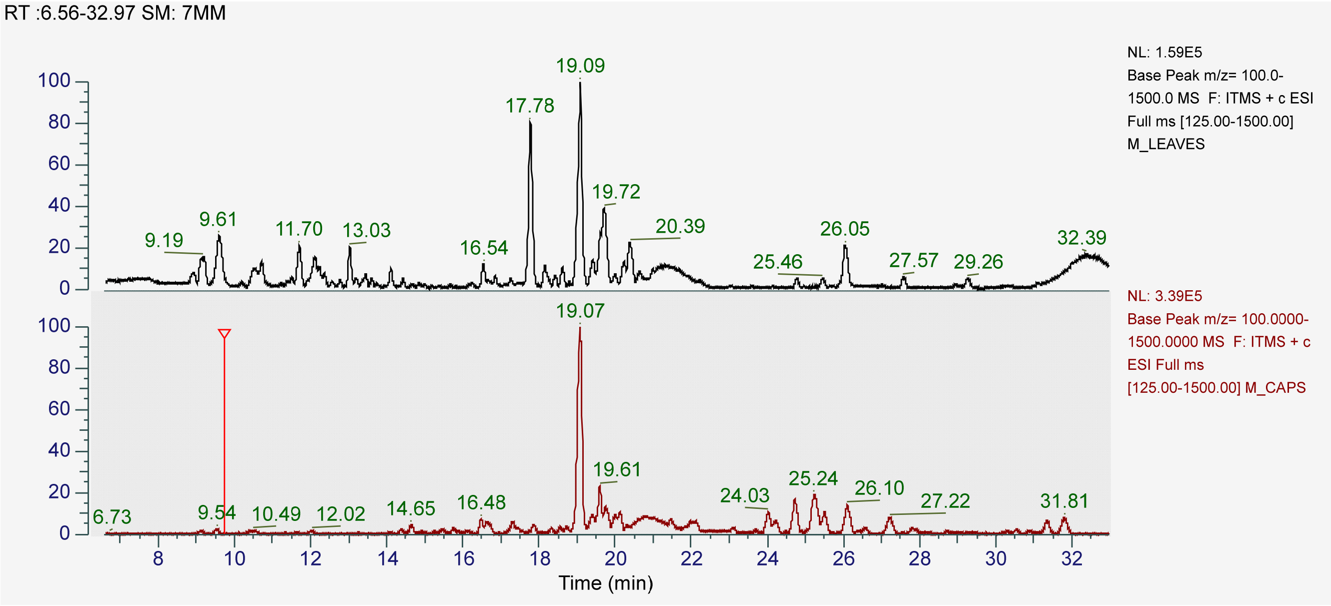

LC-MS TIC chromatogram (m/z 100 – 1400), Leaves and Tablets extract.

kfmrc-tfs@kau.edu.sa
TFSKFMRC

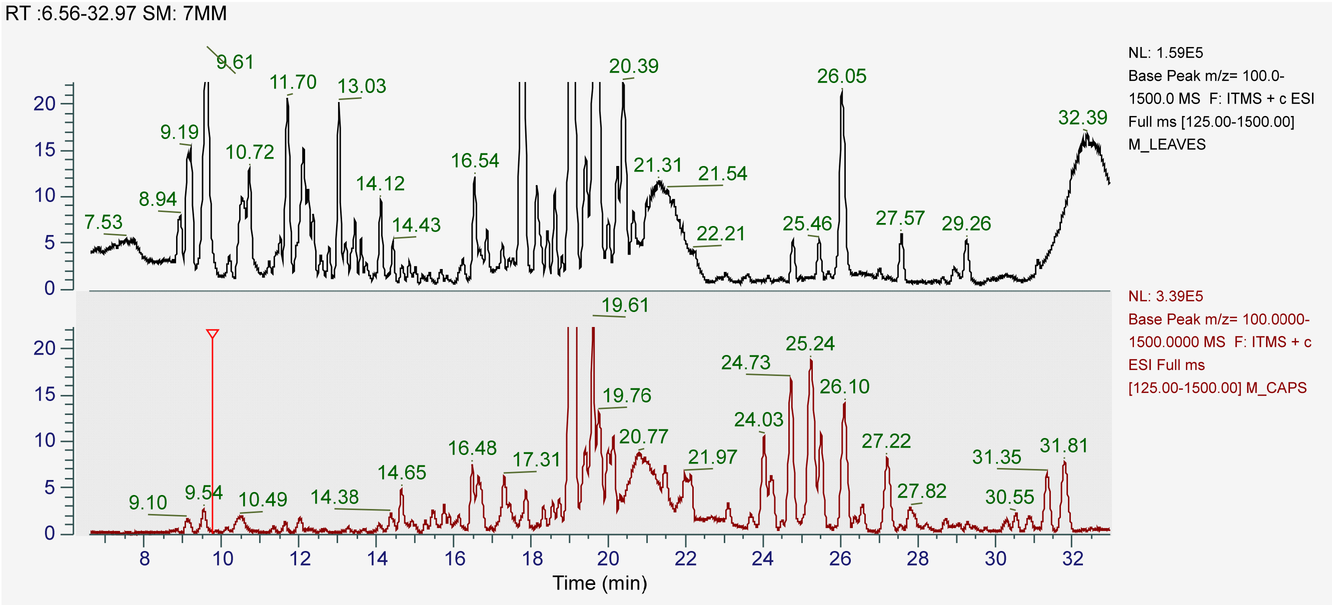

(Zoomed) LC-MS TIC chromatogram (m/z 100 – 1400), Leaves and Tablets extract.

Cucurbitacin I
Precursor ion, *m/z* 497

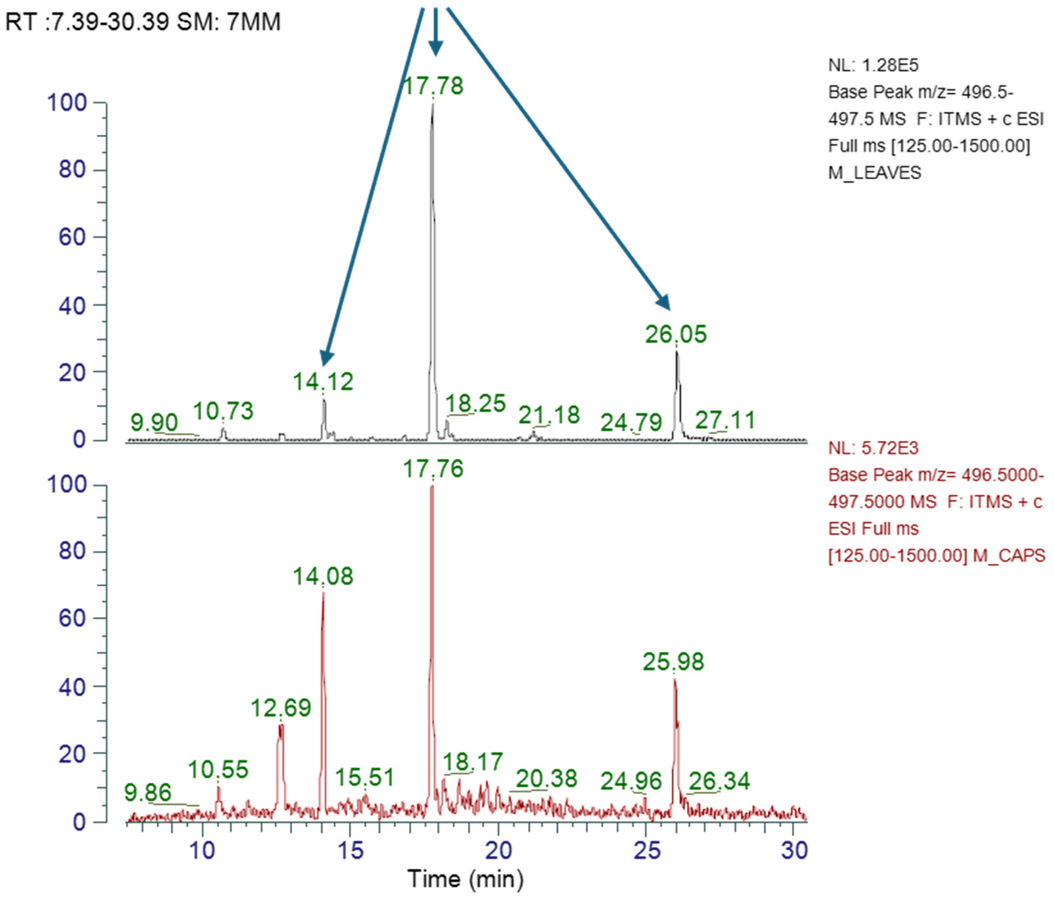

Extracted Ion Chromatogram (**EIC**) of *m/z* 497 the characteristic precursor positive ESI ion of Cucurbitacin compounds in leaves and tablets extract.

kfmrc-tfs@kau.edu.sa
TFSKFMRC

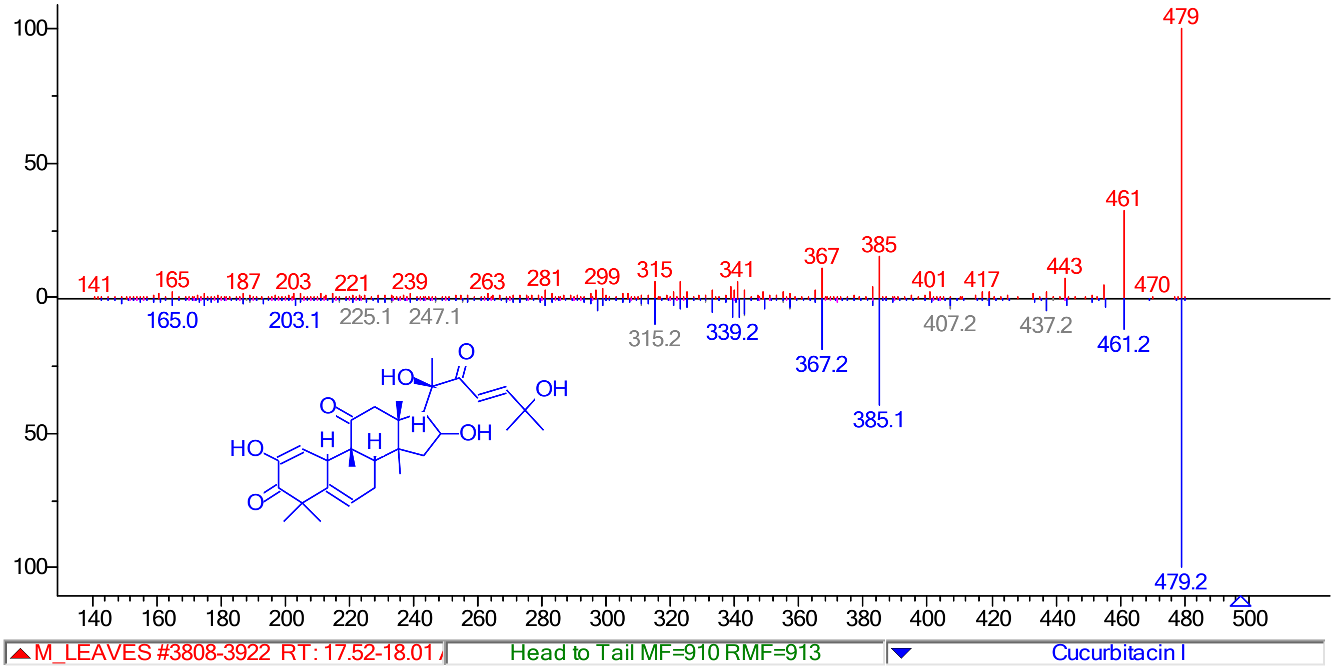

MS2 spectrum of Cucurbitacin I in the extracted sample (red) matched with that in the NIST20 database (blue)

Bacoside A-X,
Precursor ion, *m/z* 455

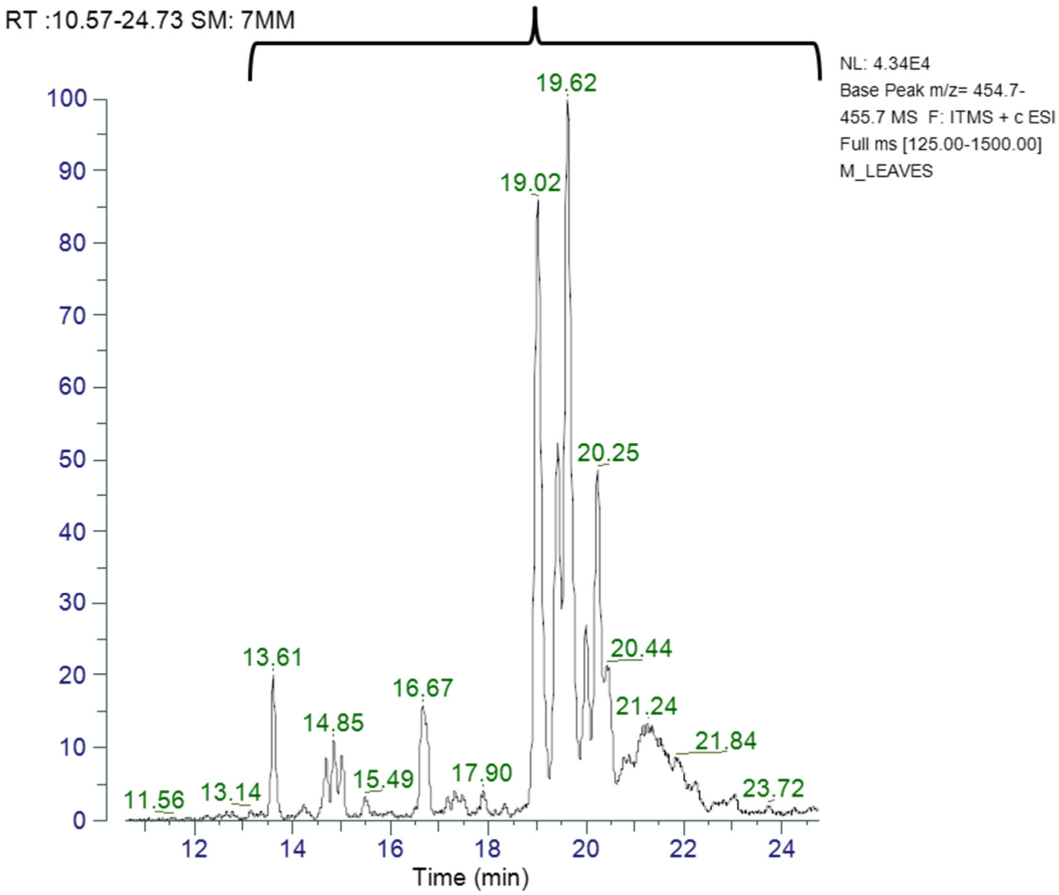

Extracted Ion Chromatogram (**EIC**) of *m/z* 455, the characteristic precursor positive ESI ion of Becoside A to X compounds in leaves extract.

kfmrc-tfs@kau.edu.sa
TFSKFMRC

RT :10.57-24.73 SM: 7MM

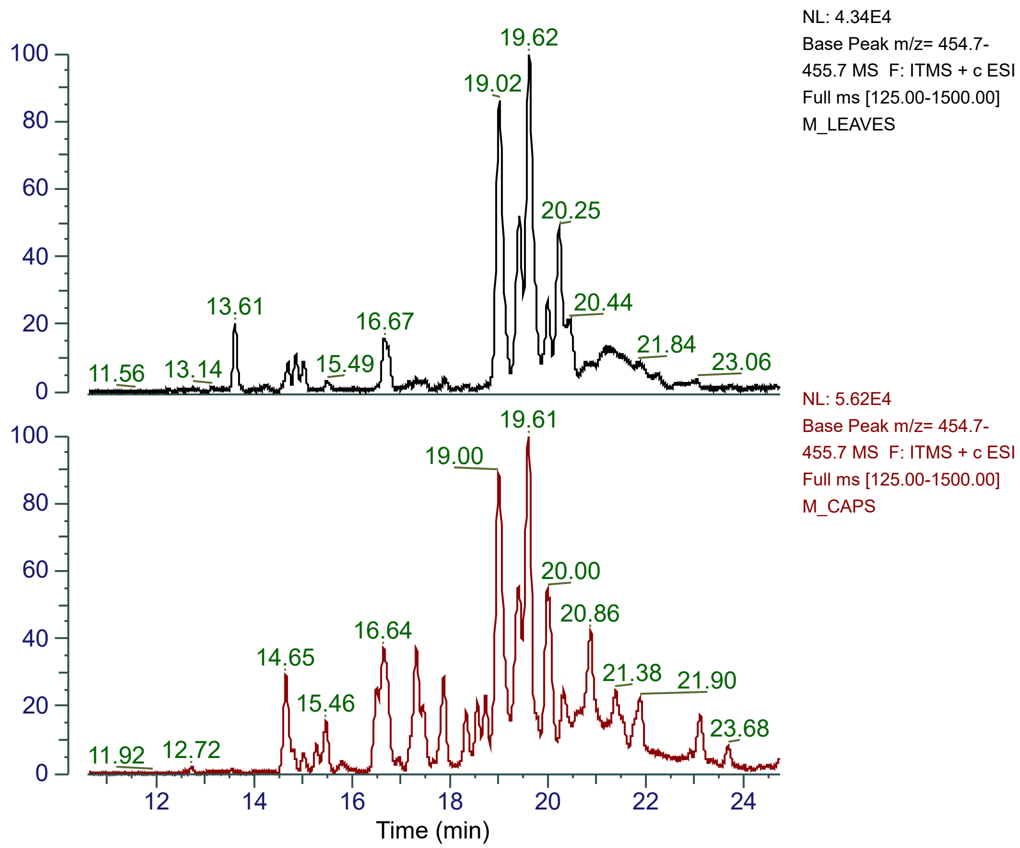

Extracted Ion Chromatogram (**EIC**) of *m/z* 455, the characteristic precursor positive ESI ion of Becoside A to X compounds in leaves and tablets extract.

Other MS2 spectra of the tabulated compounds are shown in the Excel file.

END OF REPORT

BY, Prof. Alaa Khedr

kfmrc-tfs@kau.edu.sa
TFSKFMRC
